# Metabolomic analysis of the endometrium of Large White and Meishan pigs reveals differences in biological processes during late gestation

**DOI:** 10.1186/s12864-025-12276-4

**Published:** 2025-11-21

**Authors:** Agnes Bonnet, Alyssa Imbert, Laure Gress, Nathalie Marty-Gasset, Annabelle Meynadier, Cécile Canlet, Justine Bertrand-Michel, Nancy Goeffre, Nathalie Vialaneix, Cécile MD Bonnefont, Laurence Liaubet

**Affiliations:** 1 GenPhySE, Université de Toulouse, INRAE, INPT, ENVT, Castanet Tolosan, 31326 France; 2 INRAE, Université Clermont Auvergne, Vetagro Sup, UMRH, Saint-Genes-Champanelle, 63122 France; 3 Toxalim (Research Centre in Food Toxicology), Toulouse University, INRAE UMR 1331, ENVT, INP-Purpan, UPS, MetaToul-AXIOM Platform, National Infrastructure of Metabolomics and Fluxomics: MetaboHUB, INRAE, Toulouse, 31027 France; 4 Lipidomic, MetaboHUB-MetaToul, National Infrastructure of Metabolomics and Fluxomics, Toulouse, France; 5 I2MC (Institut des Maladies Métaboliques et Cardiovasculaires), Université de Toulouse, Inserm, Université Toulouse III - Paul Sabatier (UPS), Toulouse, France; 6 Université de Toulouse, INRAE, UR MIAT, Castanet-Tolosan, 31326 France; 7 Plateforme Biostatistique, Genotoul, Toulouse, France

**Keywords:** Perinatal survival, Feto-maternal interface, Endometrium, Maternal genome, Piglets

## Abstract

**Background:**

In pigs, genetic progress has led to an increase in perinatal mortality, mainly due to reduced piglet maturity. The end of gestation (90-110 days of gestation) plays a decisive role in the acquisition of fetal maturity. The endometrium is crucial for its acquisition as it provides nutrients to the fetus through the placenta. The aim of this study is to describe part of the metabolism of the endometrium in late pregnancy and in relation with neonatal survival. For this purpose, we performed untargeted metabolomic measurements by 1H nuclear magnetic resonance and gas chromatography coupled to a flame-ionization detector on 224 endometrial samples and compared the two days of the end of gestation (D90-D110, term at 114 days) and two maternal breeds with contrasted birth survival: Large White (LW, higher birth mortality) and Meishan (MS, lower birth mortality).

**Results:**

Out of the 191 metabolites available in the ASICS package reference library, 46 metabolites and nine neutral lipids were quantified in the endometrial samples.

Twenty-two metabolites showed a significant differential abundance in the endometrium between 90 and 110 days of gestation. These differences highlighted a decrease in the amount of glucogenic amino acids such as aspartate, glutamate, and glutamine at D110, indicating a depletion of energy resources in the endometrium. As a result of hypoxic catabolism to maintain energy levels, hypoxanthine and succinate accumulated, possibly contributing to the regulation of hypoxia, ROS and modulation of inflammation of the endometrium during late pregnancy.

The study also documented ten endometrial metabolites with a significant differential abundance between LW and MS sows. Glutathione metabolism metabolites showed a decreased abundance in LW, which may contribute to increased oxidative stress. Furthermore, the accumulation of glutamine and phenylalanine may be a possible response to lower amino acid availability in LW, inducing more cellular autophagy and lower maternal immune tolerance in LW endometrium compared to MS.

**Conclusions:**

For the first time, these data provide a metabolic status of the endometrium during late gestation and between two extreme breeds for piglet survival. They reinforce the role of succinate, glutamine, and phenylalanine in influencing piglet survival birth.

**Supplementary Information:**

The online version contains supplementary material available at 10.1186/s12864-025-12276-4.

## Background

Over the past few decades, the carcass quality and reproductive traits, such as fertility and prolificacy, have become some of the most genetically and economically important traits in pigs. As a consequence, genetic selection has led to an increase in both litter size and perinatal mortality, as well as within-litter variation in prenatal development and in birth weight [1]. The comparison of newborn piglets issued from Large White boars born either in 1977 or 1998 showed less body protein and energy content, lighter liver weight and less liver glycogen stores for the 1998 newborns [2]. These variations lead to heterogeneity in postnatal growth and to high variation in weight at weaning [1, 3]. For example, low birth weight piglets were associated significantly with reduced average daily gain during suckling, post-weaning periods and meat quality [4]. Numerous factors have been demonstrated to exert influence on piglet survival both at birth and in the early postnatal period [5, 6]. The factors under consideration are as follows: the sow, including intrauterine conditions, farrowing duration, parity, and overall health; the piglets themselves, including genetic background and vitality at birth; characteristics partly shaped by maternal effects, including birth weight or a combination of these elements. A plethora of studies have repeatedly emphasized that birth weight, hypothermia, and the delay in first suckling whether considered in isolation or in combination are the most critical determinants of survival, primarily due to their association with starvation or crushing. It has been hypothesized that piglet maturity is a relevant indicator of survival and postnatal growth [7, 8]. Maturity at birth, defined as the complete development necessary to survive at birth [5, 6, 9], reflects the outcome of intricate intrauterine growth and maturation processes that occur during the final stages of gestation. Indeed, a reduced maturity at birth is observed in highly selected breeds, such as Large White compared to a more rustic Meishan (MS) [6–12] even at the molecular level in muscle [7, 9], in the intestine [8], and in fluids (urine, plasma, and amniotic fluid [10]) in late gestation. The rustic MS produces piglets with significant low mortality (proportion of stillborn piglets per litter: 3% (± 1.3 SE) for MS; 6.5% (± 0.5 SE) for LW), despite these piglets being lighter at birth (1.32 kg (± 0.05 SE) for MS and 1.51 kg (± 0.04 SE) for LW) [11].

In pigs, and in most mammals, the last third of gestation, approximatively 90 days of gestation to birth, corresponds to the maturation of numerous organs and tissues that need to be fully functional at birth [8, 9, 13, 14]. During this period, the nutrient demand increases sharply to achieve fetal maturation and growth, which places a nutritional burden on the dam. Achieving the right balance between the nutrients required for optimal fetal growth and maturation as well as the nutrients necessary for the mother’s well-being is crucial to support gestation and ensure healthy litters [15, 16]. This balance occurs at the feto-maternal interface, which refers to the interaction between the uterus/endometrium of the mother and the placenta of the fetus.

In late pregnancy, close to term, the uterine endometrium undergoes drastic changes in the expression of many genes and functions in preparation for parturition [17]. Hormonal changes, such as a decrease in progesterone levels and an increase in estrogen levels, promote uterine contractility, gap junction formation, and increased uterine responsiveness to oxytocin and prostaglandin [18].

Despite the importance of these mechanisms, many studies of the porcine endometrial tissue were performed during the peri-implantation period. During this period, immune tolerance and feto-maternal communication has been investigated [19]. Some other recent studies have focused on the middle to end of gestation [20–23]. Particularly, they have identified differential protein expression in the endometrium at mid-gestation between Meishan and Duroc breeds. These proteins appear to be involved in metabolic pathways, such as arginine metabolism, and may influence the development process of the endometrium [20]. Several endometrial genes and miRNAs involving in the response to estradiol and influencing angiogenesis were also highlighted, which may affect the size and efficiency of the placenta [23].

To the best of our knowledge, except for endometrial dysfunction associated with endometriosis in humans [24, 25], there have been no previous studies of metabolomics and lipidomics in the endometrium during late pregnancy. However, many metabolic pathways have been investigated in the conceptus and the uterus during the peri-implantation period of gestation in livestock [26]. In pigs, in particular, a review described the metabolic pathways involved in the conceptus, placenta and uterus, focusing mainly on the importance of the glycolysis, the pentose phosphate pathways, TCA cycle (tricarboxylic acid cycle), the metabolism of glutamine and many others [27]. Nevertheless, metabolomic profiling in the uterus of fertile and infertile women undergoing assisted reproductive techniques [28] or in women with infertility associated with the Mediterranean diet [29] have shown that metabolomics is a powerful tool to unravel uterine health and gain insight into endometrial functions.

In this context, the present study aimed to provide a description of molecules involved in the metabolic chemical reactions of endometrial tissue in order to understand the role of the endometrium during the maturation process of late gestation. Moreover, by comparing the metabolomic differences between the two breeds, it is hoped that we will gain important insights into metabolic pathways that could be important for perinatal survival. Indeed, sow characteristics such as piglet survival but also litter size and birth weight were recorded during the first parity studied after birth from the same sows [30]. In this study, piglet survival in lactation (days one to seven) was significantly different between the two breeds (79.9% in LW sows and 93.5% in MS sows in average). The average litter size was 14.9 (± 0.7) for LW sows and 12.7 (± 0.7) for MS sows at birth [30].

To this end, we analyzed the metabolome using 1H nuclear magnetic resonance (NMR) and the neutral lipid profile using flame ionization detector (FID) gas chromatography in endometrial fragments adjacent to the fetus in LW and MS sows from the second parity at two stages of late gestation: 90 and 110 days.

The study allowed us to identify a depletion of glucose and glycogen in the endometrium at the end of gestation. It also revealed a decrease of some glucogenic amino acids. In addition, it showed the accumulation of hypoxanthine and succinate at D110 compared to D90. Compared to MS, the endometrium of LW accumulated glutamine and phenylalanine, while the abundance of some ‘glutathione metabolism’ metabolites decreased.

## Methods

### Experimental design

#### Sow breeding

The breeding of the sows was previously described in Girardie et *al* [30]. Twenty-eight of these sows were used for a second parity in order to study the endometrium at two days (90 and 110) of the third part of gestation. Details on animal resources and genetic design can also be found in Voillet et *al* [7, 9], and Yao et *al* [8]. Briefly, the samples came from two maternal genotypes (MG): 14 Chinese MS and 14 European LW sows reared at the GenESI experimental farm (10.15454/1.5572415481185847E12). The LW and MS sows, which had already had one litter, were inseminated with a mixture of semen from one MS boar and one LW boar. The mixture of semen was prepared using equal volumes of each semen. In total, three mixtures of semen were used to inseminate an equal number of MS and LW females. With regard to the 28 sows used in the current experiment, mixture number 1 was used to inseminate two LW and two MS sows; mixture number 2 was used to inseminate six LW and eight MS sows; and mixture number 3 was used to inseminate six LW and four MS sows. Thus, each litter consisted of purebred and crossbred fetuses: LL and ML fetuses in LW sows, and MM and LM fetuses in MS sows. Both breeds of sows received the same gestation feed in the same quantities during gestation. The average litter size was 14.9 (± 0.7) for LW sows and 12.7 (± 0.7) for MS sows [30] at birth in the same experiment but during the first parity studied after birth from the same sows inseminated with the same mixed semen of two boars (one LW and one MS). With regard to the 28 sows from the second parity at 90 or 110 days of gestation that constituted the current experiment, the observed litter size was found to be significantly different between the two breeds (17.8 ± 0.56 SEM and 14.8 ± 0.56 SEM in the LW and MS sows, respectively).

#### Sample collection

Samples were collected at two days of gestation (DG) to analyze the endometrium at the end of gestation: at 90 or 110 days, named as D90 and D110 (parturition 114–115 days in sows). The gestation length was previously documented for both breeds at the same experimental farm than ours and showed no statistically significant variation (Estimates from a mixed model: 113.3 days for MS and 113.7 for LW) [11].The sows of each breed were anaesthetized with a combination of isoflurane, propofol, zoletil and nalbuphine, and then underwent caesarean section to remove the fetuses. The sow’s anaesthesia was monitored using the following parameters: vaginal temperature, heart rate, oxygen saturation (as measured by a pulse oximeter) and respiratory rate. Then, the sow was euthanized by injection of a lethal dose of potassium chloride (KCl) at the end of the caesarean section and the uterine tract was removed. These protocols were similar to those implemented and validated by Leenhouwers et *al* [5] in a study, on 46 litters, examining the fetal development of piglets shortly before term (111 days), which involved comparing different genetic types. The fetal position within the uterus was established by numbering fetuses located from the tubal to cervical end within each uterine horn.

After laparotomy of the sow, all the fetuses were euthanized, weighed, sexed, and fetal measurements were recorded. The fetuses were genotyped to discriminate the crossbred fetuses from the purebred fetuses and to identify the four fetus genotypes: LL, LM, ML, and MM [31]. For each fetus, a juxtaposed pre-determined area (middle part between the two ends of the placenta excluding distal parts) composed of the two adjacent tissues (placenta/endometrium) was dissected. As previously described by Enders et *al* [32], the pig placenta is epitheliochorial. Consequently, the trophoblastic and endometrial epithelia are juxtaposed, with no invasion of the trophoblast into the endometrium. Placenta and endometrium tissues were carefully separated with flat-tipped forceps, immediately frozen in liquid nitrogen and stored at −80 °C. Hence, each endometrium sample and each placenta sample were associated with their juxtaposed fetus [31]. In this paper, only the endometrium tissue was investigated. The details of the experiment and sample collection are described in FAANG database (https://data.faang.org/api/fire_api/samples/INRAE_SOP_COLOcATION-tissues_20210817.pdf).

#### Sample selection

A total of 224 fetuses were selected from the 28 sows (14 LW and 14 MS sows) at two gestational stages (90 and 110 days) to account for the heterogeneity of pure LW fetuses (LL) in LW sows and crossbred fetuses (LM) in Meishan sows, considering the four fetal genotypes and the two sexes. As pure Meishan (MM) and crossbred (ML) fetuses were less heterogeneous, the number of fetuses was lower. The 224 endometrial samples derived from these 224 fetuses. We called “condition” DG for days of gestation, MG for maternal genome. The number of replicates per condition is given in Table 1. The sampling selection varied between six and ten endometrial samples per LW sows and between four and fourteen endometrial samples per MS sows.

**Table 1:** Description of the experimental design

sow breed	LW	LW	MS	MS
fetal genotype	LL	ML	MM	LM
D90 Total	42	15	14	41
F	21	8	7	20
M	21	7	7	21
sow number	7	5	5	7
sire number	3	3	3	3
D110 Total	46	12	12	42
F	24	7	4	20
M	22	5	8	22
sow number	7	7	5	7
sire number	3	2	2	2
Total	88	27	26	83

### Proton nuclear magnetic resonance (^1^H NMR) based metabolomics

#### Sample preparation

The 224 samples of endometrium were pulverized under liquid nitrogen using a MM400 Mixer Mill (Retsch, Haan, Germany) and randomized for multiple effects: DG, MG, fetal genotype, and fetal sex to avoid technical bias.

For each sample, 100 mg of frozen powder was collected under liquid nitrogen in a 2 ml FastPrep tube containing Lysing Matrix S (MP biomedicals, Irvine, CA, USA). Then 335 µl of methanol was added to each tube to quench the metabolism and avoid lipid oxidation and it was frozen at −80 °C until extraction. The extraction was performed on ice and 1170 µl of cold ultrapure water was added to each tube. The mixture was homogenized by 2 homogenization cycles of 5 min at 30 Hz on the Retsch MM400 (rack previously set at −20 °C). Then 10 µl of homogenate was transferred to a protein LoBind microtube (Eppendorf, Hamburg, Germany) for later protein quantification, stored at −20 °C until Bradford assay. Cold methanol (950 µl) and cold ultrapure water (800 µl) were added in each homogenate tube, vortexed for 10 seconds. Then 1.25 ml of cold dichloromethane was added and the mix vortexed for 10 seconds once again. The tubes were centrifuged at 2,870 g at 4 °C for 15 min.

The lower organic phase, containing hydrophobic compounds, was transferred (800 µl) into a Pyrex tube and stored at −80 °C until neutral lipids analysis with FID-gas chromatography.

2 ml of the upper aqueous phase containing hydrophilic compounds were collected in a polypropylene tube and stored at −20 °C prior to evaporation. Evaporation of the aqueous phase was performed with a vacuum concentrator (Concentrator Plus, Eppendorf, Germany). After evaporation to dryness, 450 µl of phosphate buffer prepared in deuterated water (0.2 M, pH 7.0) and containing sodium trimethylsilylpropionate (8 mg TMSP for 100 ml) as internal standard, were immediately added to each tube, vortexed for 5 min, transferred to a 1.5 ml Eppendorf tube and stored at −20 °C until nuclear magnetic resonance (NMR) analysis. On the day of the NMR analysis, the extracts were thawed, centrifuged at 2,870 g for 15 min at 4 °C and 200 µl were transferred into a 3 mm RMN tube using TECAN liquid handler robot.

#### ^1^H NMR analysis

Metabolomic ^1^H NMR spectra of the 224 endometrial samples were acquired at 300 K on a Bruker Avance III HD NMR spectrometer (Bruker BioSpin, Wissembourg, France) operating at 600.13 MHz for proton resonance frequency, using the Carr-Purcell-Meiboom-Gill (CPMG) spin-echo pulse sequence at the MetaToul-AXIOM platform. The samples were randomized to avoid a bias effect. A total of 256 transients were collected in 64 k data points. Spectrum preprocessing (solvent suppression, apodization, Fourier transformation, zero-order phase correction, internal referencing, baseline correction, and window selection) was performed using the package PepsNMR (version 1.8.1) (embedded in the R package ASICS), with the TSP peak for internal reference. Before quantification, all the spectra were aligned using the joint alignment procedure implemented in the R package ASICS (version 2.6.1) and described in Lefort et *al* [33].

Finally, the metabolites in the NMR spectra were identified and quantified using the ASICS joint quantification procedure. This used the default reference library provided in the package, which included 191 NMR spectra of pure samples, and the default parameters. The only exception was the threshold under which a signal is considered noise (noise.thres), which was set to 0.01 to make peak identification less stringent. A dataset of the quantity of 191 pure metabolites (in columns) for the 224 fetuses (in rows) was obtained.

Finally, a clean-up threshold was set to 20%. This means that only the metabolites identified (non zero quantification) in more than 80% of samples for at least one condition were kept in the list of the identified metabolites, as recommended in the joint quantification procedure of [33]. A total of 46 metabolites were utilized for the following analyses.

### Lipid analysis with FID gas-chromatography

The lipid fraction of metabolomic extractions was analyzed for nine neutral lipids by gas chromatography coupled to a Flame-Ionization Detector (GC-FID) in a subset of 192 endometrium. The subset was selected from the original 224 endometrium samples with the aim of maintaining the same distribution of samples by sex, fetal genotype and sow than the original dataset and overcoming heterogeneity within different fetal genotypes. The repartition of replicates per condition for lipidomic analysis is given in Supplemental data 1. Briefly, the hydrophobic phase (800 µl), previously described in the sample preparation section, was evaporated to dryness, dissolved in 160 µl of ethylacetate, transferred into the glass insert of a 2 ml chromatography vial (Agilent Technologies, USA). Then, 100 µl of an internal standard mix was added (stigmasterol (6 µg), cholesteryl heptadecanoate (6 µg), glyceryl trinonadecanoate (16 µg)) and a last evaporation step was performed to dryness. Lipids were dissolved in 100µl of ethylacetate and stored at −20 °C until GC-FID analysis.

The lipidic extract (1 µl) was analyzed with a GC TRACE 1300 (Thermo Electron) system and a Zebron ZB-5MS Phenomenex column (5% polysilarylene, 95% polydimethylsiloxane, 5 m x 0.25 mm internal diameter, 0.25 μm film thickness). Oven temperature was programmed from 190 °C to 350 °C at a rate of 5 °C/min and the carrier gas was hydrogen (5 ml/min). The injector and the detector were at 315 °C and 345 °C, respectively.

The quantifications of the nine neutral lipids (Cholesterol, Cholesterol C16, Cholesterol C18, Cholesterol C20) and five triglycerides (TAG: TAG48, TAG50, TAG52, TAG54, TAG56) were expressed relative to the internal standard specific to each class and then normalized by tissue weight. This method did not detect cholesterol C20 or TAG48 in the endometrium, either because these metabolites were absent or because their abundance was below the lower limit of sensitivity of the assay.

### Protein measurement

The protein concentration of each endometrium sample homogenate previously stored at −20 °C was determined by Bradford protein assay (BIO-RAD, Hercules, CA 94547, USA), according to the protocol supplied by the manufacturer.

### Statistical analysis

All statistical analyses were performed with R (version 4.3.0) [34].

#### Multivariate analyses

A Principal Component Analyses (PCA) was performed (using a function from the R package ASICS) to detect potential outliers and batch effects due to experimental covariates: sex, position in endometrium and sow.

Orthogonal Projections to Latent Structures Discriminant Analyses (OPLS-DA) [35] was also performed (using a function available in the R package ASICS) to identify the metabolites with the highest discriminant power between the two DG and the two MG. The most influential metabolites, i.e. metabolites with a VIP index greater than 1, were extracted. The relevance of the results was insured by estimating the predictive power of each model with a 10-fold cross-validation error.

#### Mixed models

Mixed models were used to identify metabolites with differential concentrations between conditions (DG (days of gestation) and MG (mother genotypes)), by fitting the following model for each metabolite:

1 $${\mathrm Y}_{\mathrm{ijk}}\;=\;\mathrm\mu\;+\;{\mathrm{DG}}_{i\;}+\;{\mathrm{MG}}_{j\;}+\;\mathrm{Iij}\;+\;{\mathrm S}_{\mathrm k}\;+\;{\mathrm\varepsilon}_{\mathrm{ijk}}$$

with y_ijk_ the vector of metabolite concentrations for DG_i_ (i ∈ {D90, D110}), MG_j_ (j ∈ {LW, MS}), and the sow k. In this model, µ is the mean effect, DG_i_ the fixed effect of the DG, MG_j_ the fixed effect of MG, I_ij_ the effect of the interaction between DG and MG, S_k_ ~ N(0, σ^2^_s_) the random effect of the sow and ε_ijk_ ~ N(0, σ^2^_e_) a noise term. The mixed models were fitted with the R package lme4 (version 1.1–27.1) [36]. The analysis controls for the mother effect, minimizing variation that could arise from differences among sows with taking the sow as random effect S_k_. This model was tested against the reduced model with only the sow effect (y_ijk_ = µ + S_k_ + ε_ijk_) with a Fisher test. *P*-values were adjusted with the Benjamini and Hochberg correction [37] for controlling False Discovery Rate (FDR) in multiple testing.

Then, each differential metabolite (FDR < 0.005) was associated with one of the following sub-models as in [7] by.

2 $$\begin{array}{l}\mathrm{complete}:\;{\mathrm b}_{\mathrm{ijk}}\;=\mathrm\mu\;+\;{\mathrm{DG}}_{\mathrm i}\;+\;{\mathrm{MG}}_{\mathrm j}\;+\;\mathrm{Iij}\;+\;{\mathrm S}_{\mathrm k}\;+\;{\mathrm\varepsilon}_{\mathrm{ijk}}\\\mathrm{additive}:\;{\mathrm b}_{\mathrm{ijk}}\;=\;\mathrm\mu+{\mathrm{DG}}_{\mathrm i}+{\mathrm{MG}}_{\mathrm j}+{\mathrm S}_{\mathrm k}\;+\;{\mathrm\varepsilon}_{\mathrm{ijk}}\\\mathrm{only}\;\mathrm{stage}:\;\;{\mathrm b}_{\mathrm{ijk}}\;=\;\mathrm\mu+{\mathrm{DG}}_{\mathrm i}+\;{\mathrm S}_{\mathrm k}\;+\;{\mathrm\varepsilon}_{\mathrm{ijk}}\\\mathrm{only}\;\mathrm{genotype}:\;\;{\mathrm b}_{\mathrm{ijk}}\;=\;\mathrm\mu+{\mathrm{MG}}_{\mathrm j}+\;{\mathrm S}_{\mathrm k}\;+\;{\mathrm\varepsilon}_{\mathrm{ijk}}\end{array}$$

Since the four models described above are not nested, we used a model selection approach and selected the model with the minimum Bayesian Information Criterion (BIC) [38].

### Biological analysis for metabolites

Ingenuity^®^ Pathway Analysis software (IPA; http://www.ingenuity.com) was used to examine the connectivity between differential metabolites and to identify functional enrichment in cellular functions and canonical pathways. The significance of the association between the differential metabolites lists and functional categories was determined by a *p*-value calculated using Fisher’s exact test. Then, *p*-values were adjusted using the Benjamini and Hochberg correction for controlling False Discovery Rate (FDR) in multiple testing. The IPA results were considered significant when the FDR was less than 0.05 or the log₁₀(FDR) was greater than 1.3. A Z-score provided by IPA was used to suggest an activation state (positive Z-score) or an inhibition state (negative Z-score) by using information about the direction of metabolites regulation. The IPA results were considered when they were statistically significant (FDR < 0.05) and, as additional information, when an activation Z-score was predicted.

In this study, the core analysis module of the IPA platform was used to obtain 1) the signaling pathways significantly affected by differentially quantified metabolites using the IPA’s “Canonical pathway” module; 2) the significant biological functions and their predicted activity (activation, inhibition) using the IPA’s “Diseases and Biofunctions” module and, 3) predicted upstream regulators that could explain the changes observed in the dataset using the IPA’s “Upstream Regulator analysis” module. The analyses were performed including all the identified metabolites. Significant metabolites were associated with their log2-fold change (log2(FC) for the D110/D90 comparison or the LW/MS comparison. A log2(FC) value of 0 was assigned to non-significant metabolites to confirm their presence in pathways. This prevents them from introducing bias into predicted regulations. To identify the biological processes influenced by the change of metabolite abundance, four biological function-based networks were constructed, two with positive Z-score and two with negative Z-score, suggesting activated/inhibited biofunctions at D110 compared to D90, or LW compared to MS respectively. The metabolic networks proposed by IPA were simplified to keep the smallest connected network (in terms of edges and nodes) including all differential metabolites.

### Data integration

The integrative analysis was performed on the seven lipids and on the 46 metabolites identified in the endometrium.

A sPLS-DA was applied to metabolomic dataset to optimize the number of components and selected metabolites using cross-validation (CV). The perf function was used for CV with 10 folds and 40 repeats, resulting in the selection of two components. Another CV procedure was also run using the tune function (dist = “mahalanobis.dist” and measure = “BER”, balance error rate) to select the number of features for each component (keepX). The keepX values for further integrative analysis were 6 and 10 for the two first latent components for metabolites.

Then, data integration was performed using a sparse multiblock Partial Least Squares - Discriminant Analysis (mbsPLS-DA, also referred to as DIABLO – Data Integration Analysis for Biomarker discovery using Latent cOmponents [39]) implemented in the R package mixOmics. A first mbsPLS-DA model was carried out on endometrial lipidomic and metabolomic data to select the most discriminant variables that best discriminated the maternal genotype (LW and MS) on the first latent component. Then, two other mbsPLS-DA models were carried out by sow breed to explore the effect of the day of gestation (D90 and D110). For each mbsPLS-DA model, as the number of the lipid dataset is only seven, the number of lipids to be retained per component was chosen arbitrarily to optimize the discrimination of the conditions.

The results were visualized with the plotIndiv function, which displays the projection of individuals in the obtained latent space and allows to assess how good the discrimination of the conditions is. The plotLoading function was also used to display the contribution of the selected features attached to the components of the latent space.

### Data access

Metabolomic data including raw, quantified and filtrated metabolites data files have been deposited in MetaboLigths [40] at EMBL-EBI under accession number MTBLS3091. Lipidomic data is available at 10.57745/Q6XWHJ.

## Results

The present study aims to describe and better understand endometrial metabolism at the end of gestation (D90 and D110) in two breeds known to differ in piglet mortality (LW and MS).

### ^1^H-NMR metabolomics quantification

Of the 191 metabolites available in the ASICS package’s reference library, 46 were identified in the endometrial samples and selected for further analysis as they were present in at least 80% of at least one condition. The resulting dataset, named “metabolite data”, includes the relative quantification of 46 metabolites for the 224 endometrial samples and was made available at www.ebi.ac.uk/metabolights/MTBLS3091.

### Multivariates analysis of metabolites

**Fig. 1:**
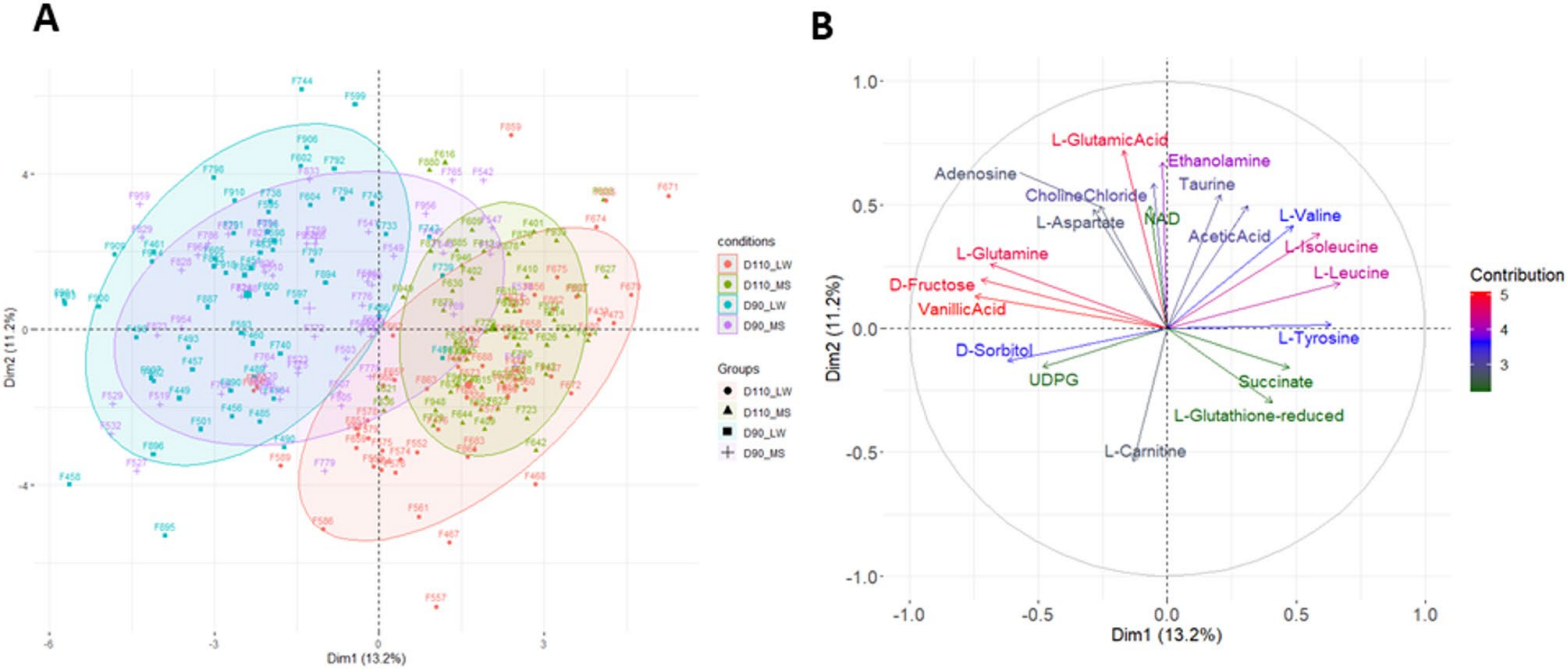
Principal component analysis of metabolite dataset. **A** Sample projection colored by MG and DG conditions, and (**B**) representation of the correlation circle with the 20 variables that were the most associated with the two first axes.The ellipses have been added to highlight the conditions of interest (the level of the ellipse is 0.8, meaning 80% of samples are included within it)

First, PCA was performed on the metabolite dataset. To identify the main drivers of the variability of the dataset, the projection of individuals was colored according to variables of the experimental design (fetal position into the uterine horn, fetal sex and sow (see Figure S1 in the Supplemental data 2) and according to MG ⨉ DG conditions (Fig. 1).

The first axis of the PCA explained 13.2% of the variance of the dataset and exhibits a clear separation between the two days of gestation (D90 and D110) (Fig. 1A). The second axis explained 11.2% of the variability. The correlation circle (Fig. 1B) shows the 20 metabolites that were the most associated to the axes, such as L-glutamine, D-fructose, vanillic acid, and D-sorbitol, (more concentrated at D90), or L-valine, L-isoleucine, L-leucine, and L-tyrosine, (more concentrated at D110). The PCA did not identify fetal sex effects (Figure S1A in the Supplemental data 2). On the contrary, a sow effect was clearly visible (Figure S1B in the Supplemental data 2) and was included in subsequent analyses whenever possible. The second axis also showed a slight separation of the endometrial samples according to their position in the uterine horn. In particular, in cases where there were more than nine fetuses, positions 9 and ≥ 10 are in close proximity to the cervix on the second axis (Figure S1C in the Supplemental data 2). The uterine position effect highlights a possible metabolic difference in the largest litters (position 9–10 may indicate a litter size of 18 piglets and above).

**Fig. 2:**
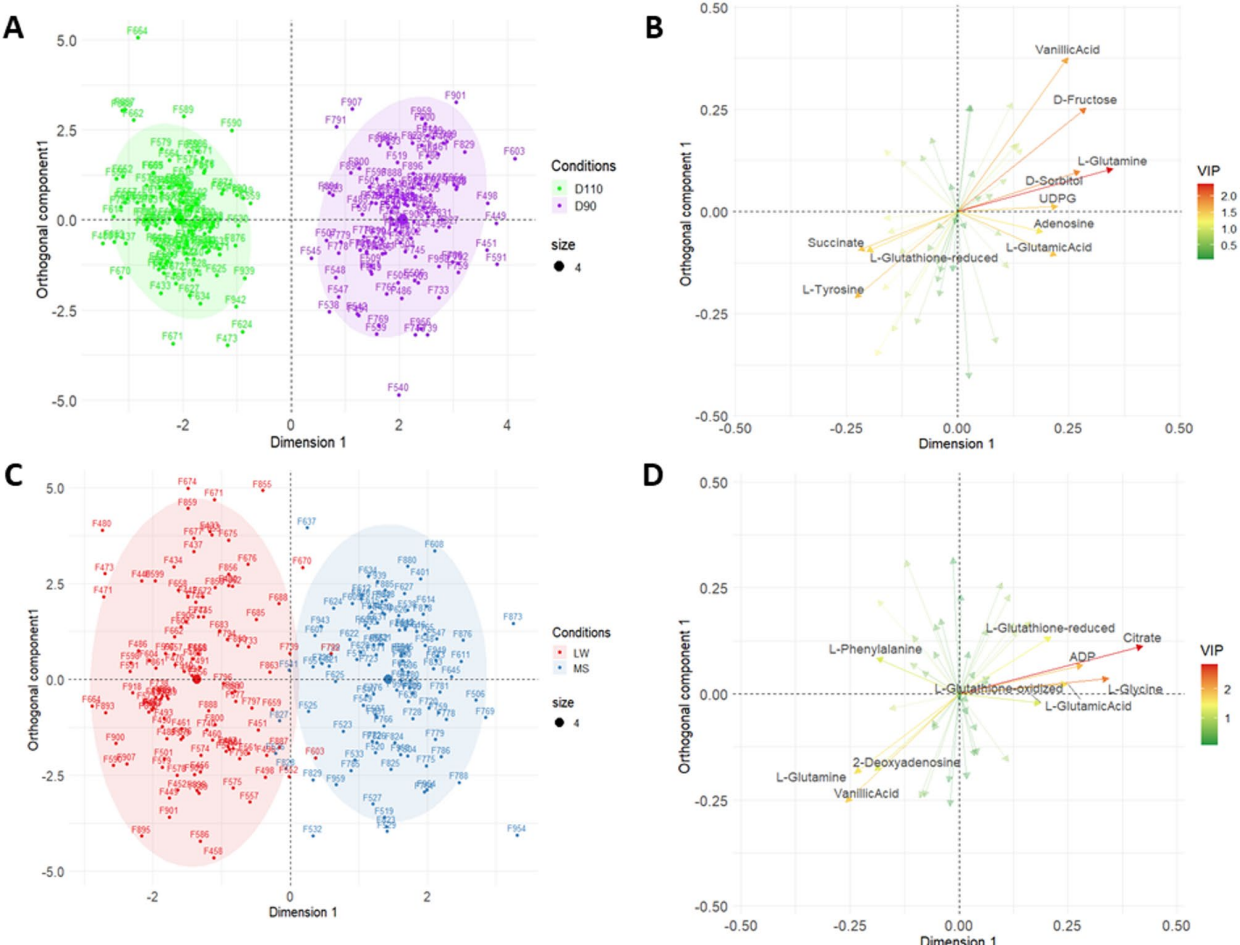
OPLS-DA visualization. Individual and variable plots for the first two axes of the orthogonal projections to latent structures discriminant analyses (OPLS-DA) on *n* = 224 endometrial samples. Figures were obtained using the quantifications from spectra for both days of gestation (D90 and D110) and the mother genotypes (LW, MS). OPLS-DA on DG: (**A**) Sample projection colored by DG, (**B**) Variable representation colored by their importance for the projection (*VIP*: Variable Influence on Projection). OPLS-DA on MG: (**C**) Sample projection colored by MG, (**D**) Variable representation colored by VIP. The ellipses have been added to better identify the conditions of interest (ellipse level = 0.95)

Two OPLS-DAs were performed separately for each factor of interest (DG and MG, Fig. 2).

The OPLS-DA on DG clearly discriminated between D90 on the right and D110 on the left (Fig. 2A). Fifteen metabolites were found to be discriminant (Variable Influence on Projection, VIP > 1, cross-validation error 0%) for DG (Table 2). For instance, L-glutamine and succinate are present in greater quantities at D90 and D110, respectively (Fig. 2B).

The OPLS-DA on MG clearly discriminated between LW on the left and MS on the right (Fig. 2C), whatever the day of gestation. Fourteen metabolites were identified as associated (VIP > 1) with the maternal genotype, with a cross-validation error of 6% (Table 2). For example, L-glutamine and citrate are present in greater and lesser quantities, respectively, in LW samples than in MS samples (Fig. 2D).

**Fig. 3:**
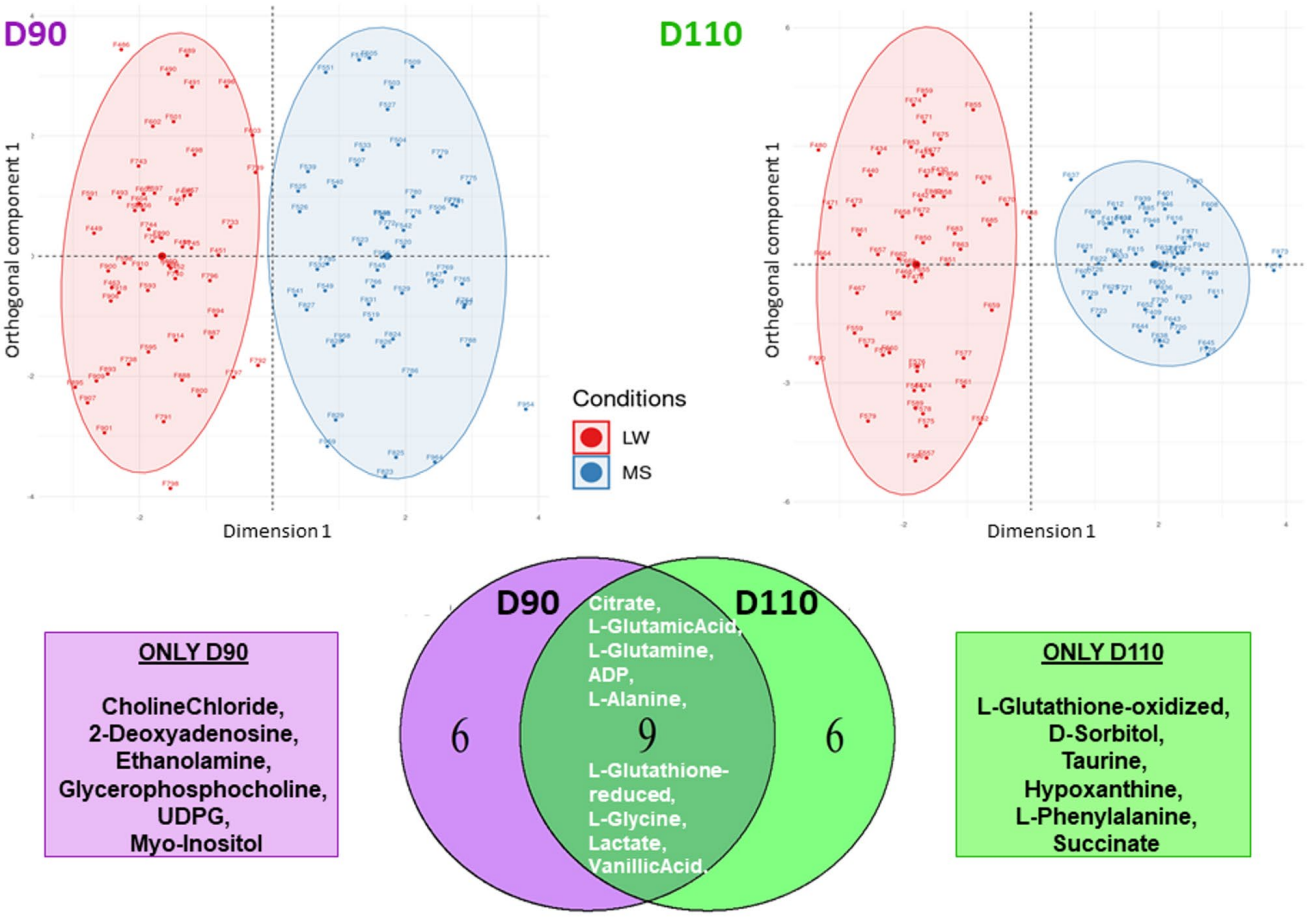
OPLS-DA for maternal genotypes. Two OPLS-DA of MG were performed separately within each DG (Left: D90 and Right: D110). The Venn diagram compares the lists of metabolites with a VIP > 1 in each OPLS-DA

Because the DG effect was strong (Fig. 1A), two OPLS-DAs were performed separately for each DG subset to better analyze the MG effect. Figure 3 shows a clear discrimination of MG (LW vs. MS) according to the first axis at each DG, with good accuracy. The cross-validation error, for both D90 and D110, was equal to 4%. The Figure S2 in the Supplemental data2 shows the variance importance plot for these two analyses. Both OPLS-DAs identified 15 discriminant metabolites for MG (VIP > 1). Nine metabolites were discriminant at both DGs and can be seen in the Venn diagram (see Fig. 3; Table 2), while six were specific to each DG.

**Table 2:** Influential metabolites for the day of gestation and the maternal genotype

Metabolite	DG VIP	MG VIP	MG at D90 VIP	MG at D110 VIP
2-Deoxyadenosine		1.3	1.34	
Acetic Acid		1.09		
Adenosine	1.3			
ADP		1.91	1.58	1.34
Citrate		2.84	2.36	2.36
Creatinine	1.12			
D-Fructose	1.96			
D-Sorbitol	1.87			1.80
Glycerophosphocholine		1.07	1.25	
Hypoxanthine	1.14	1.25		1.51
L-Glutamic Acid	1.5	1.68	1.43	2.22
L-Glutamine	2.37	1.6	2.05	1.43
L-Glutathione-oxidized		1.26		2.05
L-Glutathione-reduced	1.39	1.42	1.15	1.25
L-Glycine		2.32	2.22	1.15
L-Leucine	1.25			
L-Phenylalanine		1.28		1.29
L-Tyrosine	1.56			
Lactate	1.06		1.32	1.13
Myo-Inositol		1.1	1.03	
Phosphocholine	1.11			
Succinate	1.51			1.03
UDPG	1.52		1.13	
Vanillic Acid	1.68	1.75	1.80	1.09
Choline Chloride			1.51	
Ethanolamine			1.29	
L-Alanine			1.09	1.32
Taurine				1.58

*VIP* Variable influence on projection

### Differential analysis

**Fig. 4:**
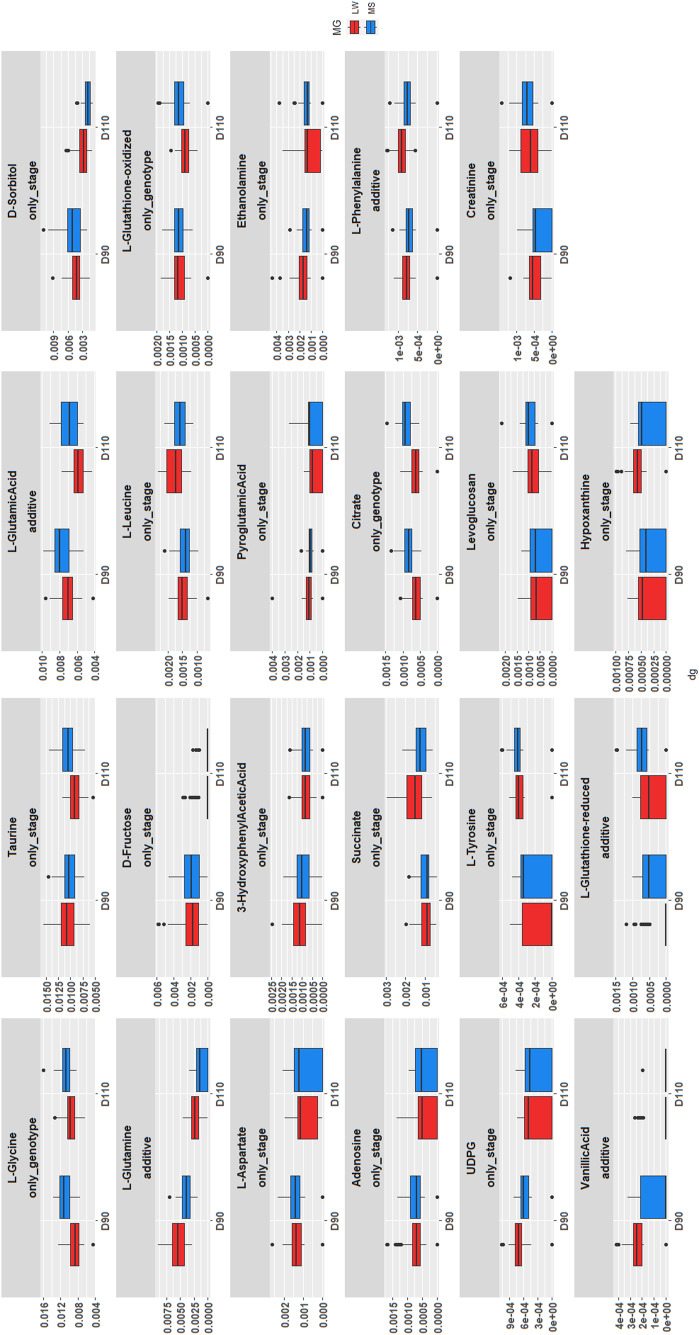
Distribution of the quantification of the 26 differentially abundant metabolites according to their DG and MG

A linear mixed model was fitted to each metabolite independently. This model included two factors (DG and MG) and their interaction as fixed effects and the sow as a random effect. After a Benjamini-Hochberg correction for multiple testing, 26 metabolites were found to be significantly differentially abundant (FDR < 0.05, Fig. 4). In a second step, these differential metabolites were associated with the best-fit sub-models derived from the full model to facilitate their individual interpretation using a BIC goodness-of-fit assessment.

Results are reported in Table 3. Twenty-two metabolites were found to be significantly associated with a model that included the effect of DG (“additive” and “only DG” models), whereas 10 differential metabolites were associated with MG (“additive” and “only MG” models). No metabolite was associated with the complete model, which indicates that the interaction effect is not of upmost importance to explain the metabolite quantifications.

**Table 3:** Number of differential metabolites associated with each sub-model

sub-model	number of metabolites
Additive (DG + MG)	6
Only DG	16
Only MG	4
All models with days of gestation	22
All models with maternal genotype	10

The distribution of the quantification for differentially abundant metabolite is provided in Fig. 4, with respect to their MG and DG. Notably, a lower amount of citrate was observed in the endometrium of LW sows compared to MS sows (see Fig. 4 and Supplemental data 3). Additionally, the study identified significant decreases in fructose and sorbitol quantification in the endometrium at the end of gestation (D110/D90; see Fig. 4 and Supplemental data 3), which is consistent with the multivariate analysis findings. Figure S3 in the Supplemental data 2 illustrates the similarities and differences between the mixed model and OPLS-DA results using a Venn diagram. For example, the OPLS-DA analysis identified all ten metabolites found to be differentially abundant for the MG effect (Figure S3B in the Supplemental data 2), demonstrating a high degree of concordance between the two methods.

All statistics from the OPLS-DAs and mixed models and their associated fold changes are summarized in Supplemental data 3.

### Functional analysis of metabolomic results

Ingenuity Pathway Analysis (IPA) was employed to investigate the biological and physiological functions of the 46 identified endometrial metabolites. IPA retained 44 of them (the S-Acetamidomethylcysteine and the levoglucosan were removed by IPA).

#### Metabolic change at the end of gestation

**Fig. 5:**
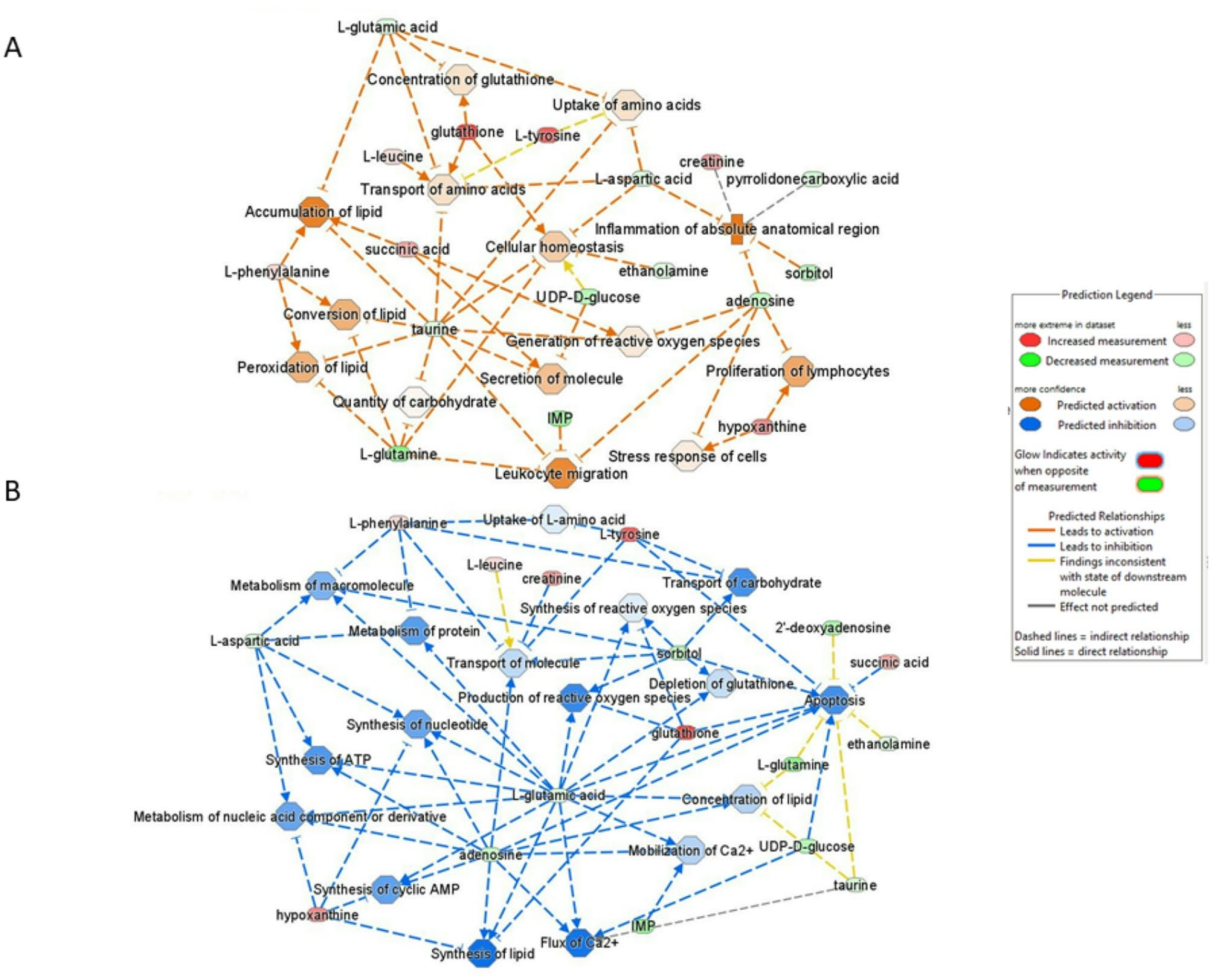
Predicted biofunction activities during the end of gestation. Networks are constructed based on the statistically significant biological functions and their trend in increase or decrease in functions between D90 and D110 (Z-score). Color scale is based on the Z-score, with orange indicating a trend in increasing state and blue indicating a trend in decreasing state. **A** Network based on predicted activated biofunctions (positive Z-score), which corresponds to a trend in increasing activity of the biological functions at D110. **B** Network based on predicted inhibited biofunctions (negative Z-score), which corresponds to a trend in decreasing activity of the biological functions at D110

The objective was to analyze the difference between D90 and D110 in order to highlight the metabolic changes that occur just prior to farrowing. We performed IPA analyses with all 44 metabolites. When significant variation in metabolic abundance was observed, the metabolites were associated with their log2 fold change (log2 FC) (D110/D90). A metabolite with non-significant variation was linked with a log2 FC of 0 (see Methods section). Top canonical pathway analyses revealed 12 pathways (FDR < 0.05) including three transport pathways (“Transport of bile salts and organic acids, metal ions and amine compounds”; “Transport of inorganic cations/anions and amino acids/oligopeptides”; “Transport of vitamins, nucleosides, and related molecules”) and four pathways (“Phenylalanine and tyrosine metabolism”; “Sulfur amino acid metabolism”; “Aspartate and asparagine metabolism”; “Glutamate and glutamine metabolism”) involved in amino acid metabolism (Table S1 in the Supplemental data 2). The analysis of diseases and biofunctions performed by IPA revealed a number of statistically significant biological functions that provide additional indications such as an increase or decrease in biofunction (positive and negative Z-scores, respectively). These results were used to construct two networks based on significant biological functions, one for each positive and negative z-score. These networks were simplified to include only differential metabolites. The first network represented the significant biofunctions, showing a predicted activation trend between D90 and D110 (Fig. 5A). It highlighted biofunctions such as inflammatory process, peroxidation of lipid, and regulation of carbohydrate metabolism at D110. The peroxidation of lipid also participates to the enriched “Ferroptosis pathway” (-log(adjusted *p*-value) = 2.66 (data not shown), to regulate cell death. The second network represented the biofunctions showing a predicted decrease trend between D90 and D110 (Fig. 5B). It highlighted biofunctions such as lipid synthesis (Z-score = 1.9), protein metabolism, apoptosis, and Ca^2+^ flux at D110. Furthermore, these two networks demonstrated the involvement of glutathione metabolism, cellular oxidative stress and cell death in the endometrium during late gestation.

 IPA also predicted significant upstream regulators that may influence the metabolite abundance at the end of gestation (FDR < 0.05). The most significant regulators suggested by IPA included metabolite regulators such as D-glucose (Figure S4A in the Supplemental data 2) and Low Density Lipoprotein (LDL, Figure S4B in the Supplemental data 2), transcription factors such as the huntingtin (HTT) involved in intracellular transport (Figure S4C in the Supplemental data 2), and matrix metallopeptidase 11 (MMP11), which may be associated with the remodeling process and angiogenesis at the maternal-fetal interface (Figure S4D in the Supplemental data 2). *HTT* and *MMP11* genes were indeed expressed in the endometrium (unpublished data that derived from [41]). In accordance with Figure S4, the glucose abundance was below the lower limit of sensitivity. Other predicted significant upstream regulators are involved in the inflammatory response (IL10, IL36 and cytokines). Details are presented in Table S2 in the Supplemental data 2.

#### Metabolic change between maternal breeds

**Fig. 6:**
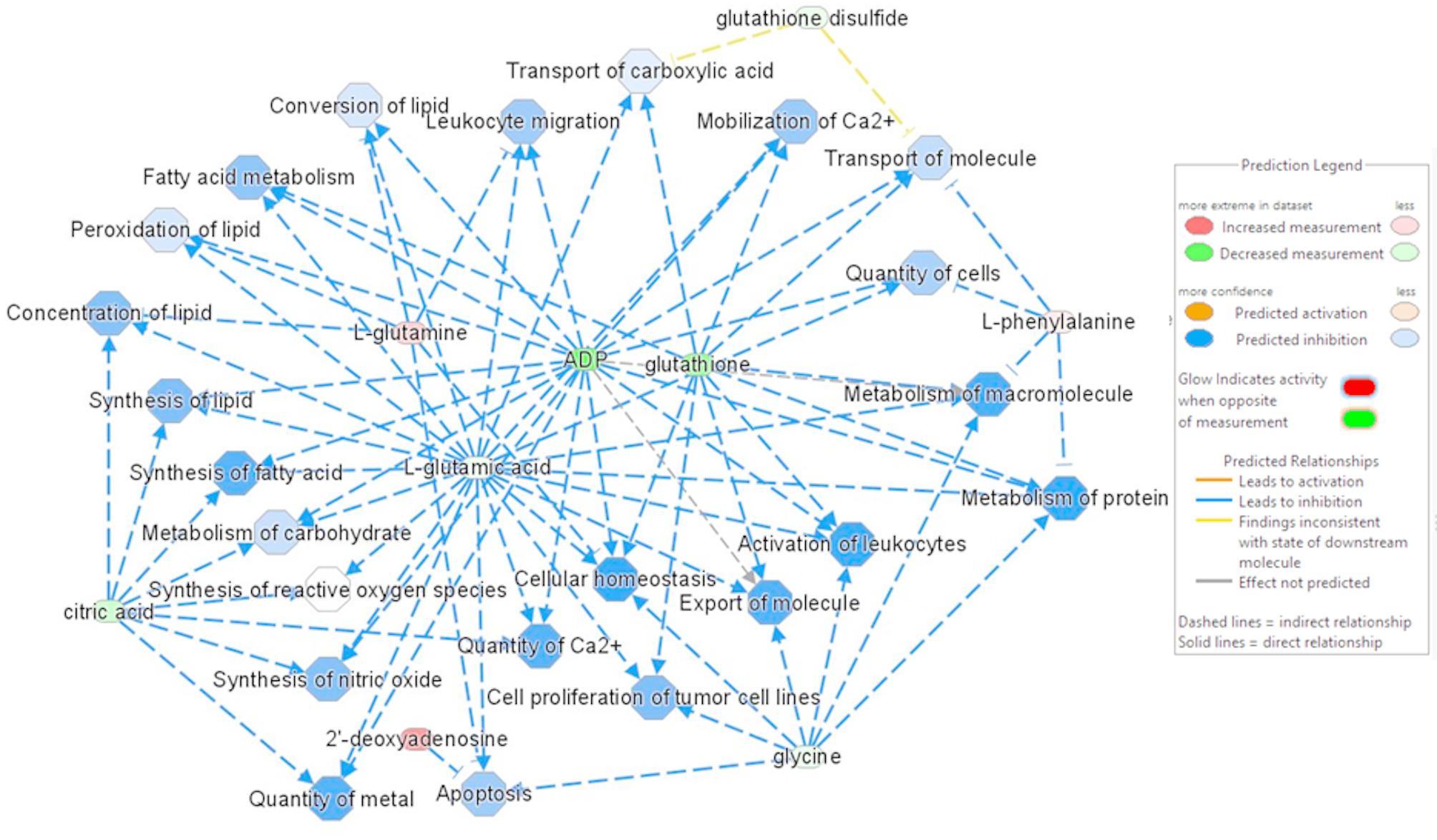
Predicted significant biofunctions in LW endometrium compared to MS. The network is based on predicted significant biofunctions that show a trend of inhibitory relationships (negative Z-score). These relationships correspond to the decreasing activity of biofunctions in LW compared to MS, regardless of gestational age

 IPA analyses were then applied to the 44 identified metabolites associated to their log2-FC (LW/MS) when observed variations were significant or associated to a log2(FC) of 0 for the metabolites with non-significant variations (see Methods section). Top canonical pathway analyses revealed 13 pathways (FDR < 0.05) that highlighted the “Ferroptosis Signaling Pathway” and eight other pathways such as “Phenylalanine Degradation IV”, “TP53 Regulates Metabolic Genes”, or “Arachidonic acid metabolism” (Table S3 in the Supplemental data 2). The IPA’s Diseases and Biofunctions analysis revealed a significant network associated with a negative Z-score. This indicates that the predicted biofunctions tend to be inhibited in LW when compared to MS (Fig. 6). Notably, decreased in protein metabolism (Z-score = −2.045) and in cellular homeostasis (Z-score = −2.121) were observed in LW compared to MS sows (Fig. 6). Other biofunctions related to lipid metabolism (z-score= −1.08) and immune processes (“Activation of leucocytes”: Z-score= −1.98) also tended to be downregulated in LW (Fig. 6).

 IPA upstream regulator analysis found significant top regulators (Figure S5 in the Supplementary data2) involved in energy metabolism, such as the regulatory-associated protein of mTOR Complex 1 (RPTOR) (Figure S5A in the Supplementary data2), and immune response, such as the regulator CD274 (Fig. 5B in the Supplementary data2). The prediction of these master regulators is reinforced by the lower expression of *CD274* gene in LW endometrium (Maman-Haddad et *al*, unpublished data; [35]) compared to MS (Figure S6A in the Supplementary data2). Finally, RPTOR forms a complex with mTOR, which showed a decreased gene expression in LW compared to MS (Figure S6B in the Supplementary data 2). The details of the IPA upstream regulator analysis are presented in Table S4 of the Supplemental data 2.

### Protein and neutral lipid quantification

Significant change in protein concentration (µg protein/mg tissue) was assessed using a Wilcoxon test. For lipid, the analyses focused on lipid class belonging to the sterol (Cholesterol, Cholesterol C16, Cholesterol C18) and triglycerides (TAG50, TAG52, TAG54, TAG56), using relative abundance. A linear mixed model was fitted to each lipid, as in the metabolomic analysis.

The protein concentration showed a significant decrease in LW endometrium at the end of gestation with 40.29 and 33.28 µg of protein per mg of tissue, respectively for D90 and D110 (Table 4; *p*-value < 0.005, Wilcoxon test), but not in the MS endometrium (*p*-value > 0.05, Wilcoxon test; Figure S7 in the Supplemental data 2).

After a BH correction for multiple testing, the relative abundance of TAG52 and TAG54 were found significant for the additive effect (DG + MG; adjusted *p*-value = 0.02 for both) and of TAG50 and TAG56 showed a tendency for additive effect (adjusted *p*-value = 0.084 for both). None lipid class belonging to the sterol was found significant (adjusted *p*-value > 0.1; Table 4). The relative abundance of the four TAGs was observed to decrease at D110 compared to D90 and to increase in LW compared to MS (Figure S8 in the Supplemental data 2).

**Table 4:** Statistics for protein concentration and neutral lipid abundance associated with each sub-model. Protein concentration (µg of protein/mg of tissue) differences between conditions were assessed using Wilcoxon test.. The adjusted *p*-value of triglycerides was obtained using the BH method. The sub-model was fitted and specified with MG for maternal genome and DG for days of gestation. The average relative abundance is reported for each lipid class. TAG (triacylglyceride) classes is followed by the total number of carbons (50/52/54/56) of the four fatty acids

	*p* value	Adjusted *p*-value	sub model	Average abundance			
				LW D90	LW D110	MS D90	MS D110
Protein concentration	0.000031	0.0002	DG for LW	40.29	33.28	36.34	35.83
Cholesterol	0.10	0.145		0.21	0.20	0.20	0.17
Cholesterol C18	0.61	0.61		0.0032	0.0026	0.0032	0.0026
Cholesterol C16	0.34	0.40		0.0012	0.0011	0.0011	0.0011
TAG50	0.042	0.084	DG + MG	0.0031	0.0024	0.0021	0.0018
TAG52	0.006	0.020	DG + MG	0.0084	0.0056	0.0050	0.0038
TAG54	0.003	0.020	DG + MG	0.0059	0.0046	0.0037	0.0030
TAG56	0.036	0.084	DG + MG	0.0020	0.0017	0.0016	0.0015

### Covariations of metabolomic and lipidomic data

Multiblock sparse partial least squares discriminant analyses (mbsPLS-DA) were used to explore the variables that best discriminated MG and DG.

**Fig. 7:**
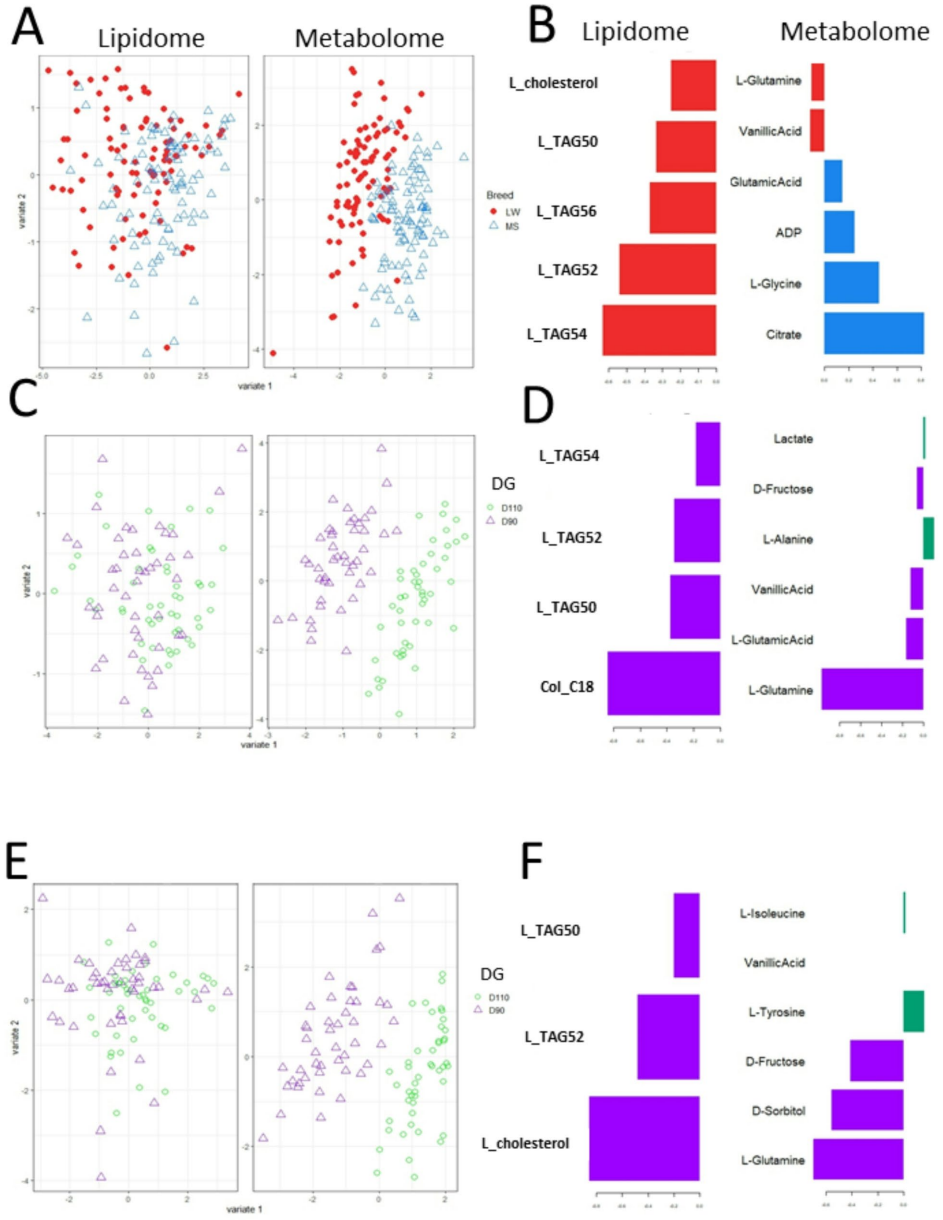
Sample projection and loading values associated with the selected discriminant variables for maternal genotypes and days of gestation from lipidomic and metabolomic integration. Three multiblock sPLS-DAs were performed on MG and DG using lipidomic and ^1^H-NMR metabolomic datasets to identify the variables that most effectively differentiate between the conditions on the first latent component. Sample projections are built with the Y matrix such as the maternal genotype (**A**) and days of gestation for Large White sows (**C**) and Meishan sows (**E**) separately. **A** Red and blue colors represent the samples from LW and MS, respectively. **C**-**E**- Purple and green colors represent the samples from D90 and D110, respectively. The plotLoadings represent the weights of the contribution of each variable to the first latent component. **B** Top discriminant lipids and metabolites for MG. **D** Top discriminant lipids and metabolites for DG in LW sows. **F** Top discriminant lipids and metabolites for DG in MS sows. For each lipid or metabolite, colors indicate the group in which the median expression is maximal

Figure 7 shows the results of the three mbsPLS-DAs performed for the MG and DG effects. For the metabolomic data, the three sample projection plots showed good separation of the studied conditions on the first latent component (Fig. 7 A, 7 C and 7E). However, for the lipidomic data, the same plots exhibited only slight separation between MG (Fig. 7 A) and no separation between DG for each maternal breed (Figs. 7C and E).

The mbsPLS loading values graphs were plotted in Fig. 7B and D F. Five lipids and ten metabolites were found to be important for the first latent component and discriminant for MG (Fig. 7B). The most discriminating variables were the TAG54 for lipids and the citrate for metabolites. For the DG effect, only the metabolite plots are informative at D90 and D110 (Fig. 7D and F). The main variable associated with the first latent component that discriminated the DG groups was the L-Glutamine at both D90 and D110.

## Discussion

The endometrium is a metabolically active tissue, with significant nutrient and energy requirements that are critical for supporting fetal growth and survival at birth. It is therefore of paramount importance to determine its metabolism in late gestation. In pig, the placenta is epitheliochorial and the placenta and endometrium are juxtaposed, with no trophoblast invasion into the endometrium, allowing the two organs to be studied with negligible contamination. To ensure nutrient transfer, the uteroplacental interface develops complex folds and areolae structures containing uterine gland secretions (histotrophic such as glucose and fructose as well as amino acids) [42, 43].

In this study, we used NMR techniques to obtain the metabolic profile of the sow’s endometrium on two key days during gestation: D90 and D110. These days are associated with the majority of fetal maturation, which takes place during the final third of gestation. We also compared two breeds: LW vs. MS, with high and low neonatal piglet mortality, respectively. As a result of fetal demand, the endometrial adaptations may differbetween MS and LW sows to optimize resource allocation and ensure fetal maturation. Our aim was to identify key differences in metabolites in late gestation. The study reported the detection of 46 endometrial metabolites among the 191 metabolites available in the ASICS package’s reference library and nine neutral lipids. It should be noted that this ^1^H NMR analysis is limited to the aqueous metabolites and to the metabolites present in the ASICS reference library. Nevertheless, the ASICS method and stringent cleanup threshold provide greater confidence in the quantification. The discussion is limited to significant metabolites.

### Metabolic status at the end of gestation

**Fig. 8:**
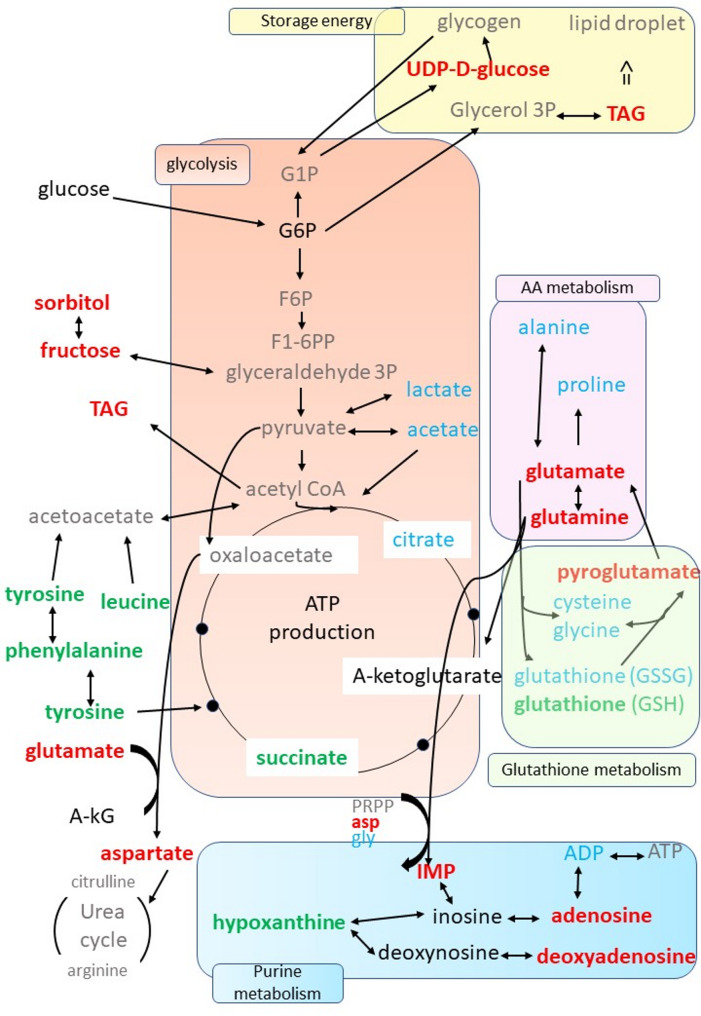
Summary of the metabolic pathways impacted during late gestation. The green color indicates the metabolites with significantly higher concentrations at D110 than at D90. The red color indicates the metabolites with significantly lower concentrations at D110 than at D90. The blue color indicates the metabolites identified by ^1^H-NMR but not differential between D90 and D110. The black color indicates the metabolites that were absent (they were present in the ASICS reference library, but their abundances were predicted to be zero). The grey color indicates the metabolites that could not be quantified because they are not present in the ASICS reference library. Abbreviations: G1P: glucose-1-phosohate; G6P: glucose-6-phosohate; G3P: glycéraldéhyde-3-phosphate; F6P: fructose-6-phosphate; F1-6-PP: fructose-1,6-biphosphate; a-KG: α-ketoglutarate; PRPP: phosphoribosylpyrophosphate; asp: asparagine; gly: glycine; IMP: inosine monophosphate; TAG: triglycerides; ADP: adenosine diphosphate; ATP: adenosine triphosphate, AA: amino acid

During late gestation, the rapid growth and development of the fetus results in the mother maintaining a high catabolic state [44]. As a result, the present study showed a decrease in the amount of protein, triacylglycerol (TAGs), and cholesterol in the endometrial tissue on D110 compared to D90 (Table 4). This finding corroborates our earlier study, wherein we demonstrated a diminution in the expression of the final enzyme in the cholesterol biosynthesis pathway (DHCR7) in the endometrium at D110 [31]. We also identified enriched metabolomic pathways involved in cellular energy processes such as carbohydrate, amino acid and glutathione metabolisms, using the 22 differential metabolites at the end of gestation. These pathways are summarized in Fig. 8.

#### Carbohydrate metabolism

The “Carbohydrate metabolism pathway” plays a critical role in fetuses at the end of pregnancy and at birth to provide the energy needed for maintenance, thermoregulation and growth after birth. At the end of gestation, high glucose levels were observed in the plasma of fetal pigs [10], though fructose was the most abundant hexose sugar [45]. Both glucose and fructose are of maternal origin and are transported to the fetus [43]. In fetuses, glucose serves as an important source of carbon for metabolic processes [46] and is converted to glucose-6-phosphate (G6P), which is the first intermediate in glucose metabolism. At the end of gestation, the sow develops reversible insulin resistance to increase glucose transport to the fetuses [47]. Consistent with this, our metabolomic analysis did not detect glucose and G6P in the endometrium at D90 and D110. The lack of detectable intracellular levels of glucose in the endometrium may be related to a greater transfer to the fetus and conversion via the alternative polyol pathway to fructose. Indeed, all the enzymes belonging to the polyol and fructose transporters are present at the feto-maternal interface in pigs [43]. Our study also found a strong decrease in the amount of fructose (D110/D90 fold change = 0.16) and sorbitol (D110/D90 fold change = 0.53) in the endometrium (Supplemental data 3). This suggests that fructose may have already been transported and accumulated in the fetus. These results are consistent with the decrease in fructose and sorbitol and increase in glucose previously observed in fetal pig plasma at the end of gestation [10]. They may explain the decrease in the energy storage in the endometrium. In fact, the abundance of four members of the TAG50 family (Table 4) and of UDP-D-glucose (UDPG) decreased between D90 and D110, and glycogen was no longer detected at the end of gestation. UDPG is a precursor for glycosylation, allowing the synthesis of glycogen, oligosaccharides and glycoproteins. The resulting glycans and glycan-binding proteins play a critical role in maternal immune tolerance [48, 49]. These results showed an altered glucose metabolism with a depletion of the energy available in the endometrium, illustrating the nutritional burden on the mother at the end of gestation.

Only a small number of metabolites involved in the glycolysis pathway were found in our study because most were not present in the ASICS reference library (i.e. they cannot be detected). Nevertheless, two tricarboxylic acid cycle (TCA cycle) metabolites were present in the porcine endometrium at the end of gestation: citrate and succinate.

#### Glutathione metabolism

Berchieri-Ronchi et *al* [50] shows that there is an increase in systemic oxidative stress in sows during late gestation, which may be caused by overproduction of reactive oxygen species (ROS) [51]. Glutathione (GSH) is known to have an antioxidant capacity that helps maintain the redox cycle of cells [52]. It also prevents premature birth and fetal death. Reduced glutathione (GSH) is formed from glutamate, cysteine, and glycine and protects cells from oxidative damage by scavenging hydrogen peroxide and hydroperoxides. The GSH/GSSG (Oxidized glutathione) couple is essential for maintaining the cellular redox state. As expected, our study shows that the “Glutathione metabolism” was enriched in differential metabolites for DG effects (Fig. 8). The concentration of endometrial GSH increased at D110. In line with this result, the concentration of pyroglutamate, an intermediate in the glutathione cycle, has already been shown to be lower at D110. Indeed, an increased concentration of pyroglutamate has been suggested as a marker of oxidative stress due to GSH depletion [53]. In addition, other metabolites such as the succinate accumulates in the porcine endometrium at D110. Succinate is an important metabolite at the junction of several pathways. In particular, succinate is involved in the homeostasis of reactive oxygen species (ROS), hypoxia response [54] and plays an important dual role as a signaling molecule during inflammatory responses [55]. Under physiological hypoxia, low oxygen levels in blood lead to succinate accumulation [56]. Succinate can also maintain hypoxia and extravillous trophoblast function and prevents recurrent spontaneous abortion [57]. Our findings indicate that succinate plays a significant role during late gestation, possibly contributing to the regulation of hypoxia and ROS. Taken together, these results are also in agreement with an increase in oxidative stress at the end of gestation [51].

#### Amino acid metabolism

The regulation of amino acid metabolism in the endometrium is one of the most important metabolic processes to meet the increased nutritional requirements of gestation and is involved in protein biosynthesis. Many studies have also shown that amino acids have an antioxidant function and can reduce oxidative stress in the body. In our study, four amino acid metabolic pathways were found to be enriched in differential metabolites at the end of pregnancy: “Phenylalanine and tyrosine metabolism”; “Sulphur amino acid metabolism”; “Aspartate and asparagine metabolism”; and “Glutamate and glutamine metabolism”. These pathways were enriched in eight different amino acids, including two essential amino acids (leucine, phenylalanine) and two non-essential amino acids (hypoxanthine, tyrosine), which were significantly more abundant at D110 than at D90, and four non-essential amino acids (taurine, glutamate, glutamine, aspartate), which were less abundant at D110 than at D90.

The amino acids of the “Aspartate and asparagine metabolism” and the “Glutamate and glutamine metabolism” (aspartate, glutamate, glutamine, taurine) have been well studied during gestation because of their essential role in fetal growth and development in pigs [58, 59]. In particular, glutamine supplementation of the sows during gestation appears to reduce the risk of fetal growth retardation in sows and to reduce pre-weaning mortality in piglets [59, 60]. Finally, glutamine is an alternative carbon source to maintain TCA cycle flux, but can also be transferred to the fetus [27]. Glutamine is also an important player in the redox balance and was identified in this study as the most discriminative metabolite for gestation days in LW and MS sows (Fig. 7C-D).

Phenylalanine and tyrosine levels are known to be influenced by oxidative stress [61]. Phenylalanine is essential for protein synthesis and serves as a precursor for important catecholamines necessary for fetal development and maternal health throughout pregnancy [62]. However, elevated maternal phenylalanine levels are toxic to the developing fetus [63]. Consistent with the findings of Wu et al. [64] who showed that the rate of phenylalanine accumulation in the whole fetal pigs increases rapidly with advancing gestation, phenylalanine also accumulates in the endometrium at D110 compared to D90. However, the phenylalanine is not present in the fetal plasma [10]. Leucine, which is more concentrated at D110, is involved in regulating protein metabolism, providing oxidative energy and enhancing antioxidant enzyme activity.

Hypoxanthine is produced by the breakdown of adenosine monophosphate (AMP) as a result of hypoxic catabolism to maintain energy levels. Under normal conditions, the majority of hypoxanthine is reutilized, whereas, in hypoxia, the rate of salvage and the degradation decreases due to energy depletion, resulting in hypoxanthine accumulation [65]. Transient physiological hypoxia has been proposed as a critical regulator of endometrial function, altering the inflammatory environment, influencing vascular remodeling and modulating endometrial proliferation [66]. The accumulation of hypoxanthine in endometrium on D110 is consistent with an increased hypoxia at the end of the gestation.

Finally, glucose, fructose, amino acids, polyamines, and many proteins present in the endometrium can be transferred to the fetus via the areolae structure [58], illustrating the regulation of amino acid and other molecular transport. With the exception of leucine, which accumulates in the endometrium and in fetal plasma at D110 [10], the amino acids such as aspartate, glutamate, and pyroglutamate exhibit decreased abundance in the endometrium and an accumulation in fetal plasma at D110 [10]. This is consistent with a transfer of these amino acids from the endometrium to the fetus as alternative substrates for feto-placental metabolism. Conversely, phenylalanine, tyrosine, and hypoxantine have been found to accumulate in the endometrium at D110, indicating a distinct function within the endometrium as they are not detected in fetal plasma [10]. This cellular and extracellular amino acid trafficking is supported by the predicted significant upstream regulator huntingtin (HTT) protein, as identified by IPA analysis (Figure S4C in the Supplemental data 2). Huntingtin is ubiquitously expressed throughout the body and plays a role in the regulation of intracellular dynamics, endocytosis, autophagy, and transcription. In particular, huntingtin mediates trafficking of vesicles and organelles [67] and its gene is expressed in the endometrium (unpublished data derived from [41]).

#### Immune-inflammatory mechanisms

A number of studies have described the importance of regulating the immune-inflammatory response at the feto-maternal interface throughout gestation [19, 68–70]. The current study illustrated these processes by showing a significant decrease in UDPG and an increase in the hypoxanthine, succinate, and phenylalanine metabolites at D110. UDPG-derived glycans and glycan-binding proteins play a critical role in maternal immune tolerance [48, 49]. As discussed above the hypoxanthine accumulation at D110 may alter the inflammatory environment of the endometrium near parturition. Succinate plays a central role in modulating inflammation [55]. Our results once again point to an important role of succinate in the late stages of gestation. Finally, intraperitoneal injection of phenylalanine in mice disrupts cytokine-based immunity and oxidative stress in the uterus, which leads to impaired embryo implantation [71]. Last, the IPA analysis highlighted the predicted significant upstream regulator MMP11 protein (Figure S4D in the Supplemental data 2). MMPs are involved in uterine placental and vascular remodeling during normal pregnancy, preterm and term labor parturition [72]. They also affect the immune system by modulating the differentiation and immune activity of immune cells, macrophage, and neutrophil recruitment [70, 73, 74].

### Metabolic change between breeds

The main differences observed between the LW and the MS endometrium concerned the protein, lipid, and the glutathione metabolism. Proteins and lipids participate to remodeling processes, immune response, oxidative processes, and energy production. The present study shows a decrease in the protein concentration in the LW endometrium at D110 compared to D90, while the protein concentration remains in the MS (Table 4). IPA analysis also predicted a significant decrease in protein metabolism in the LW (Z-score=−2,045)(Fig. 6). Conversely, the study showed a higher amount of TAGs and cholesterol in LW than in MS endometrium. These results were in accordance with a different energy reserve strategy in the LW endometrium compared to the MS endometrium.

**Fig. 9:**
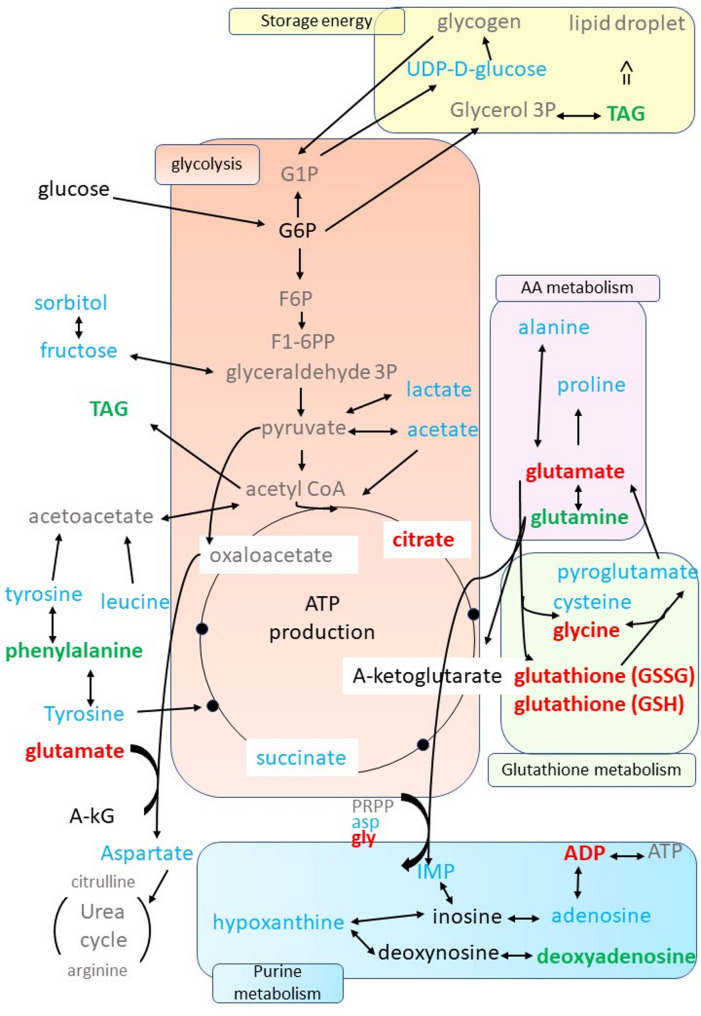
Summary of the metabolic pathways impacted by the maternal genotype (whatever DG). The green color indicates the metabolites with significantly higher concentrations in LW than in MS. The red color indicates the metabolites with significantly lower concentrations in LW than in MS. The blue color indicates the metabolites identified by ^1^H-NMR but not differential between LW and MS. The black color indicates the metabolites that were absent (they were present in the ASICS reference library, but their abundances were predicted to be zero). The grey color indicates the metabolites that could not be quantified because they are not present in the ASICS reference library

Furthermore, metabolites of “glutathione metabolism,” such as glutamate, glycine, GSH, and GSSG, are present in lower amounts in LW than in MS (Fig. 9). This difference may contribute to higher oxidative stress in LW. In agreement with our results, Lefort et *al* [10] suggested a better oxidative defence in MS than in LW. It is interesting to note that only glutamine of the “Glutathione metabolism” is more abundant in LW than in MS. This observation suggests that the metabolism of glutamine differs between breeds.

In the IPA analysis of upstream regulators, glutamine and glutamate are associated with two significant regulatory networks. These networks highlight CD274 and a predict the inhibition of RPTOR (Z-score= −2) as upstream regulators. This association is further supported by reduced gene expression of these genes in the LW endometrium compared to the MS endometrium (Figure S4 in the Supplementary data 2). The RPTOR protein forms a complex with the mTOR protein and participates in the mTOR signaling pathway. Notably, mTORC1 is closely linked to amino acid availability, with glutamine serving as a key amino acid in this context [75]. Glutamine metabolism also regulates the mTORC1 autophagy pathway, which plays an essential role in maintaining endometrial homeostasis and function [76]. In accordance with Tan et *al* ‘s study on cells [75] the significant RPTOR/glutamine regulatory network may differentially regulate the autophagy pathway in the endometrium in response to breed-specific amino acid deprivation. These studies suggested that the mTORC1 autophagy pathway is activated by the accumulation of glutamine in response to reduced amino acid availability in LW. CD274, the other upstream regulator also interacts with glutamine. The protein encoded by *CD274* is a ligand, also known as the Programmed death-ligand 1 (PD-L1), which interacts with its receptor the programmed cell death-1 (PD-1) to inhibit cytokine production and prevent autoimmunity, thereby maintaining immune homeostasis. The receptor/ligand interaction PD-1/PD-L1 (or PD-1PD-L1 pathway) plays a critical role in maintaining immune tolerance at the feto-maternal interface [77]. *CD274* expression was also significantly lower in cultured mesenchymal stromal cells from the decidua of preterm pregnancies compared to term pregnancies [78]. Last, the accumulation of phenylalanine in the LW endometrium compared to the MS endometrium may have an effect on immunity [79], as discussed in the previous section in relation to immunological and oxidative stress in the uterus [71].

Taking together, these results reported the differential abundance of 22 metabolites that participate to enriched metabolomic pathways involved in cellular energy processes such as carbohydrate, amino acid and glutathione metabolisms.

The study also reports a reduced abundance of “glutathione metabolism” metabolites in LW compared to MS that may lead to a higher susceptibility to oxidative stress in LW sows in late gestation. The accumulation of glutamine and phenylalanine suggest a possible response to reduced amino acid availability in LW and a reduced maternal immune tolerance in LW endometrium compared to MS, respectively.

This study requires further endometrial analyses to understand the potential impact of the observed endometrial accumulations on the development of the fetuses in late gestation.

## Conclusion

This study is the first to examine the metabolic status of the endometrium at the end of gestation in two breeds of pigs contrasted for piglet survival.

The end of gestation in porcine endometrium could be characterized by an altered glucose metabolism (lack of detectable intracellular glucose and glycogen), a decrease in glucogenic amino acids (aspartate, glutamate and glutamine), possibly due to a depletion of energy resources, and in more fetal transfer. The study points to an important role of succinate in the late stages of gestation, possibly contributing to the regulation of hypoxia, ROS and modulating the inflammatory environment of the endometrium. These findings provide new insights into the endometrial metabolome and illustrate the nutritional burden on the mother at the end of gestation.

Finally, the comparison between LW and MS breeds, which differ greatly in neonatal survival (high vs. low mortality at birth, respectively), highlights differences in metabolites of the “Glutathione metabolism” pathway, suggesting less antioxidant defence in the highly prolific LW breeds. The study points to the accumulation of glutamine, which is a possible response to reduced amino acid availability in LW, as well as the accumulation of phenylalanine in LW, which would have consequences for maternal immune tolerance for this breed.

These results reinforce the important roles of succinate, glutamine, and phenylalanine in fetal maturation during the final stage of gestation, a process that is essential for the survival of piglets. Furthermore, endometrial analysis using RNA sequencing technologies will be useful to validate these pathways and clarify their genomic and genetic regulation.

## Supplementary Information

Supplementary Material 1. Supplemental data 1. Description of the experimental design for lipidomic analysis

Supplementary Material 2. Supplemental data 2. Tables and Figures that complete Metabolomic results

Supplementary Material 3. Supplemental data 3. The file displays the statistics for all the 46 metabolites from the OPLS-DAs and mixed models, along with their associated fold changes. The models reported in the select_model column were attributed only to significant metabolites (FDR < 0.05). Non-significant metabolites were annotated as NA.

## Data Availability

Metabolomic data including raw, quantified and filtrated metabolites data files have been deposited in MetaboLigths [40] at EMBL-EBI under accession number MTBLS3091. Lipidomic data is available at [10.57745/Q6XWHJ](10.57745/Q6XWHJ).
