# Supplementary material for: Metabolomic analysis of the endometrium of Large White and Meishan pigs reveals differences in biological processes during late gestation
Source: BMC Genomics. 2025 Nov 21;27:11. doi: 10.1186/s12864-025-12276-4 (PMC12771944; doi:10.1186/s12864-025-12276-4)
Supplement: Supplementary file 2 — Supplementary Material 2. Supplemental data 2. Tables and Figures that complete Metabolomic results [file 12864_2025_12276_MOESM2_ESM.docx]

# Supplemental metabolomic and lipidomic analyses

### Agnes Bonnet^1^, Alyssa Imbert^2^, Laure Gress^1^, Nathalie Marty-Gasset^1^, Annabelle Meynadier^1^, Cécile Canlet^3^, Justine Bertrand-Michel^4, 5^, Nancy Goeffre^4, 5^, Nathalie Vialaneix^6, 7^, Cécile MD Bonnefont^1,^ Laurence Liaubet ^1§^

^1^ GenPhySE, Université de Toulouse, INRAE, INPT, ENVT, 31326 Castanet Tolosan, France.

^2^ INRAE, Université Clermont Auvergne, Vetagro Sup, UMRH, 63122 Saint-Genes-Champanelle, France

^3^ Toxalim (Research Centre in Food Toxicology), Toulouse University, INRAE UMR 1331, ENVT, INP-Purpan, UPS, MetaToul-AXIOM Platform, National Infrastructure of Metabolomics and Fluxomics: MetaboHUB, INRAE, 31027, Toulouse, France.

^4^ Lipidomic, MetaboHUB-MetaToul, National Infrastructure of Metabolomics and Fluxomics, Toulouse, France.

^5^ I2MC (Institut des Maladies Métaboliques et Cardiovasculaires), Université de Toulouse, Inserm, Université Toulouse III - Paul Sabatier (UPS), Toulouse, France.

^6^ Université de Toulouse, INRAE, UR MIAT, Castanet-Tolosan -31326, France.

^7^ Plateforme Biostatistique, Genotoul, Toulouse, France.


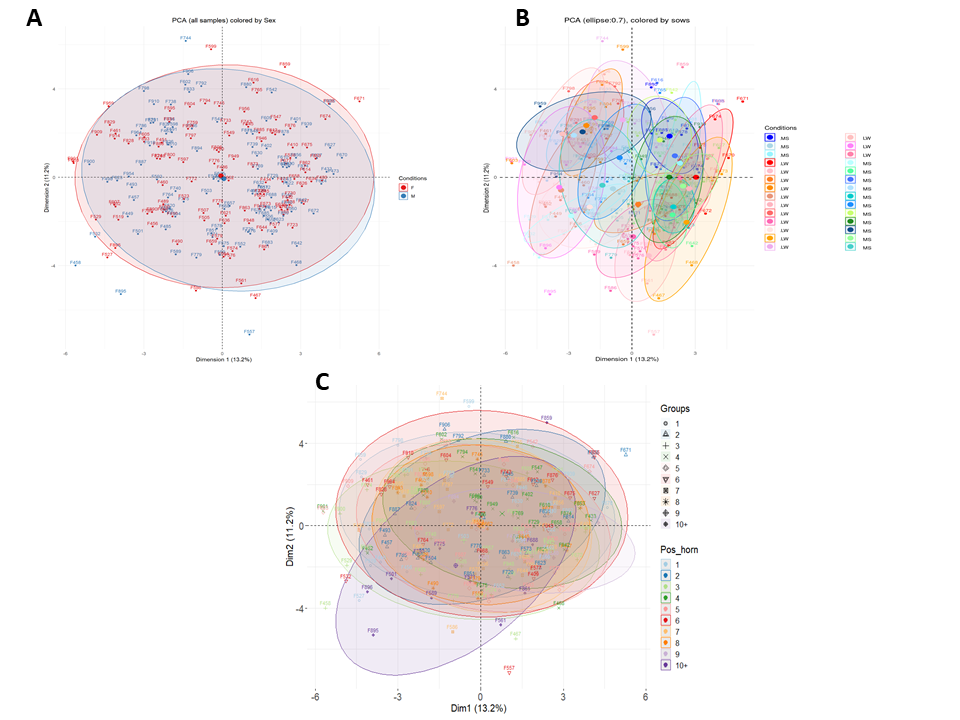


**Figure S1 - Principal Component Analysis for the two first axes**

Sample representation colored by (A) Fetal sex, (B) Sow, (C) horn position*

* The fetal position within the horn was established by numbering fetuses located from the tubal to cervical end within each uterine horn.

The ellipses have been added to highlight the conditions of interest (the level of the ellipse is 0.8, meaning 80% of samples are included within it).


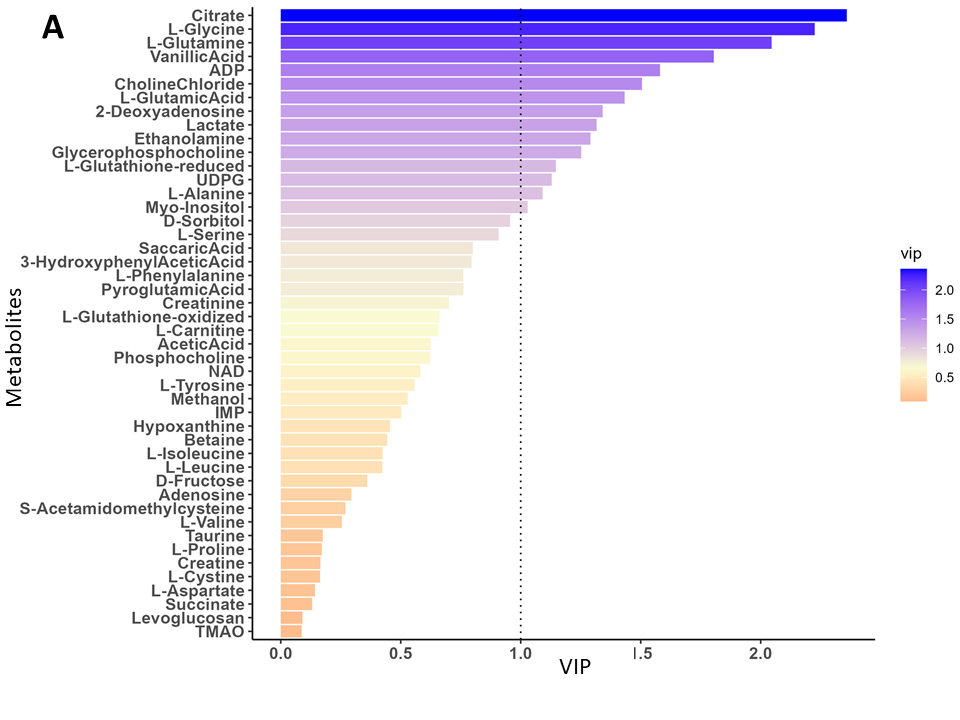

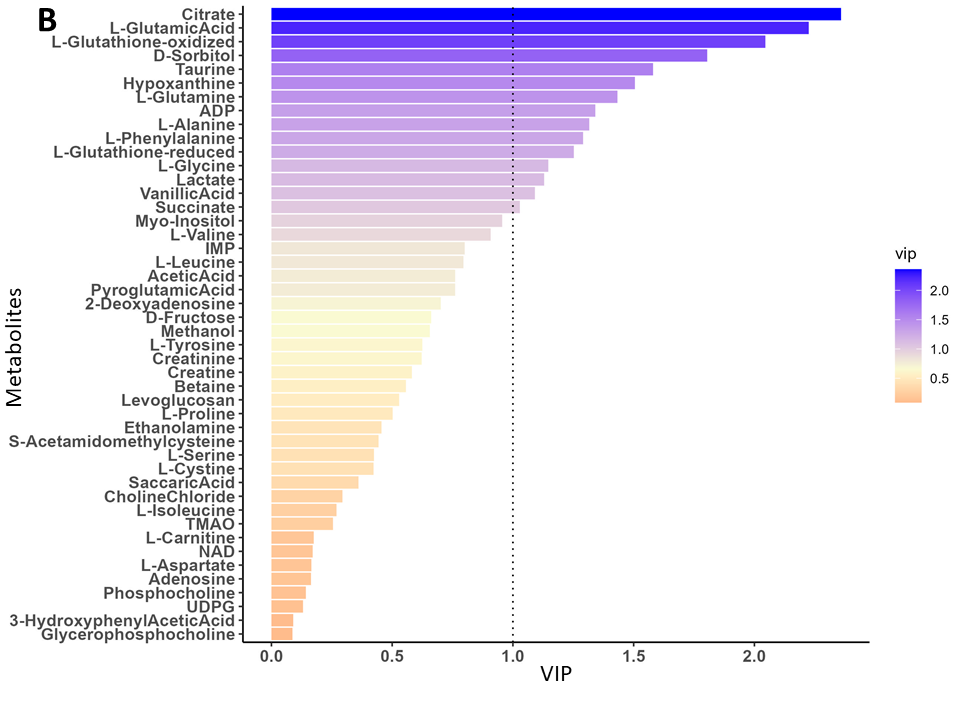


**Figure S2 –** **Variance importance plot for maternal genotype according to the day of gestation**

(A) Variance Influence for Projection (VIP) for D90,

(B) VIP for D110.


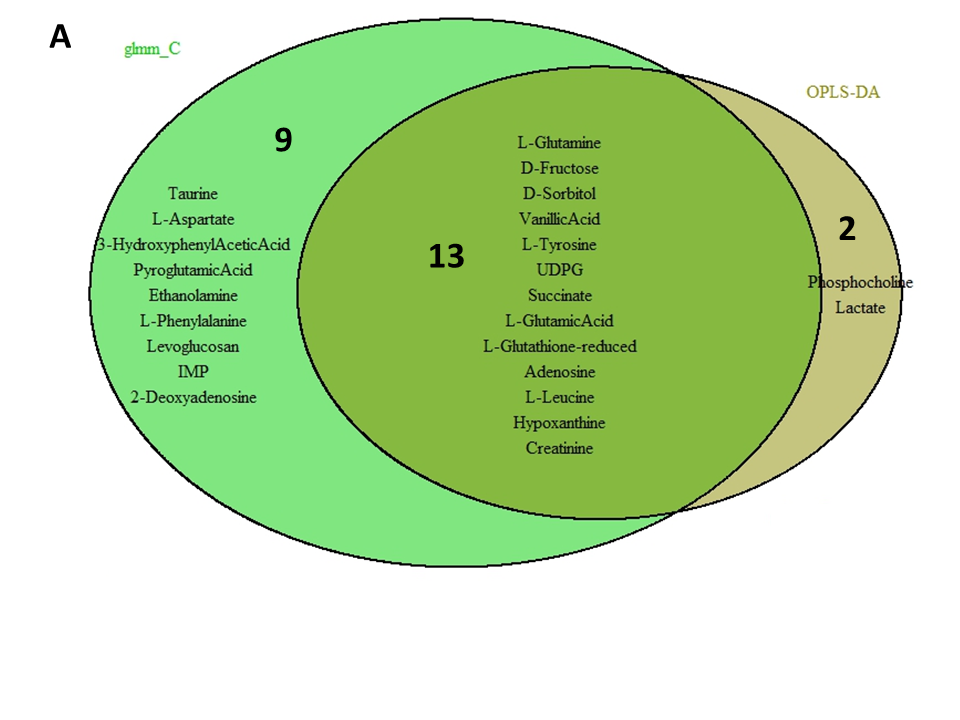


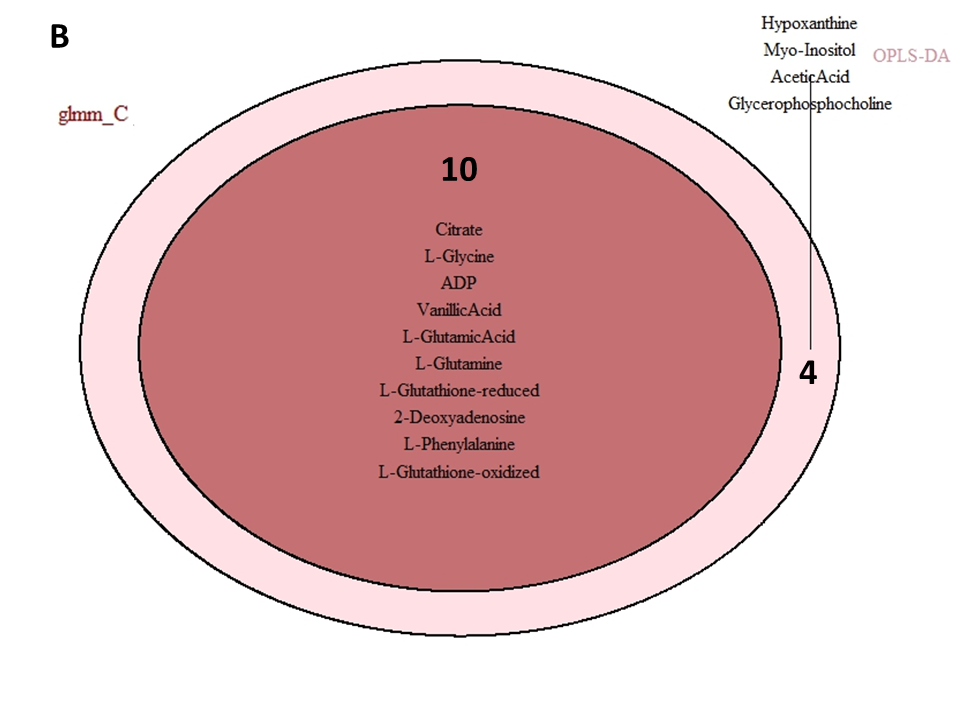


**Figure S3 – Comparison results between OPLS-DA and linear mixed models.**

(A) DG: the comparison includes 15 metabolites with a VIP > 1 from the OPLS-DA and 22 metabolites from differential analysis (16 from only DG model + 6 from additive model)

(B) MG: the comparison includes 14 metabolites with a VIP > 1 from the OPLS-DA and 10 metabolites from differential analysis (4 from only MG model + 6 from additive model).


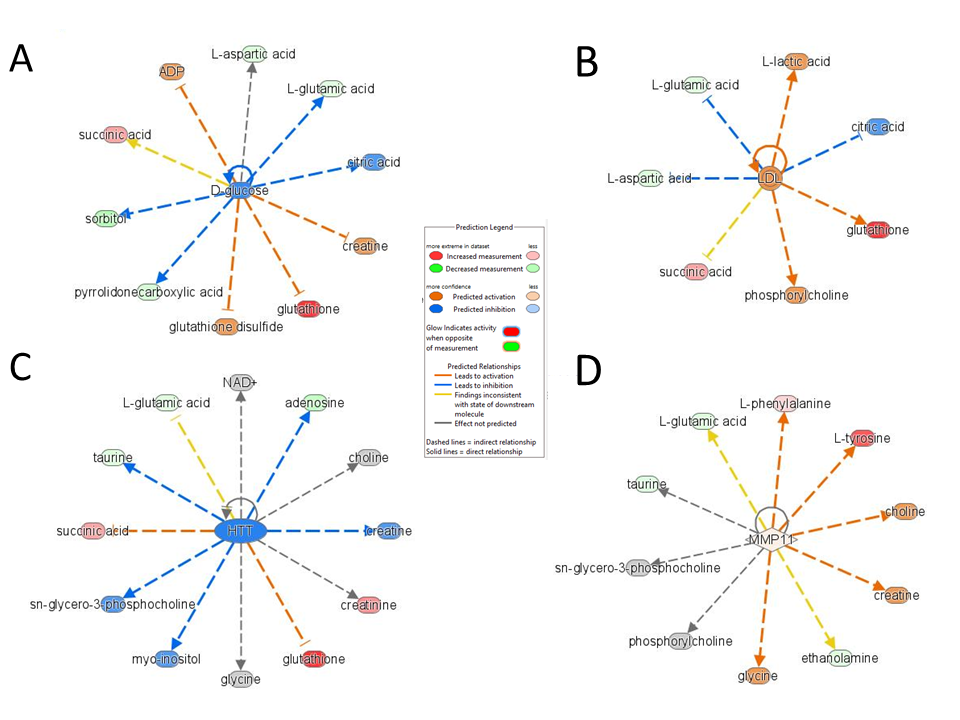


**Figure S4 – Networks of IPA's predicted significant upstream regulation for endometrial metabolites at the end of gestation.**

These networks focus on key regulators like A- glucose, B- Low Density Lipoprotein (LDL) complex, C- huntingtin (HTT), D- matrix metallopeptidase 11 (MMP11).
The pointed arrowheads represent the trend of activating relationships while the blunt arrowheads represent the trend of inhibitory relationships drawn from the available IPA literature. The blue, orange, and yellow colors indicate a predicted trend of inhibition, an activation, or an inconsistent finding of the relationship, respectively. The gray color indicates metabolites that were identified in the endometrium and did not vary between D110 and D90.


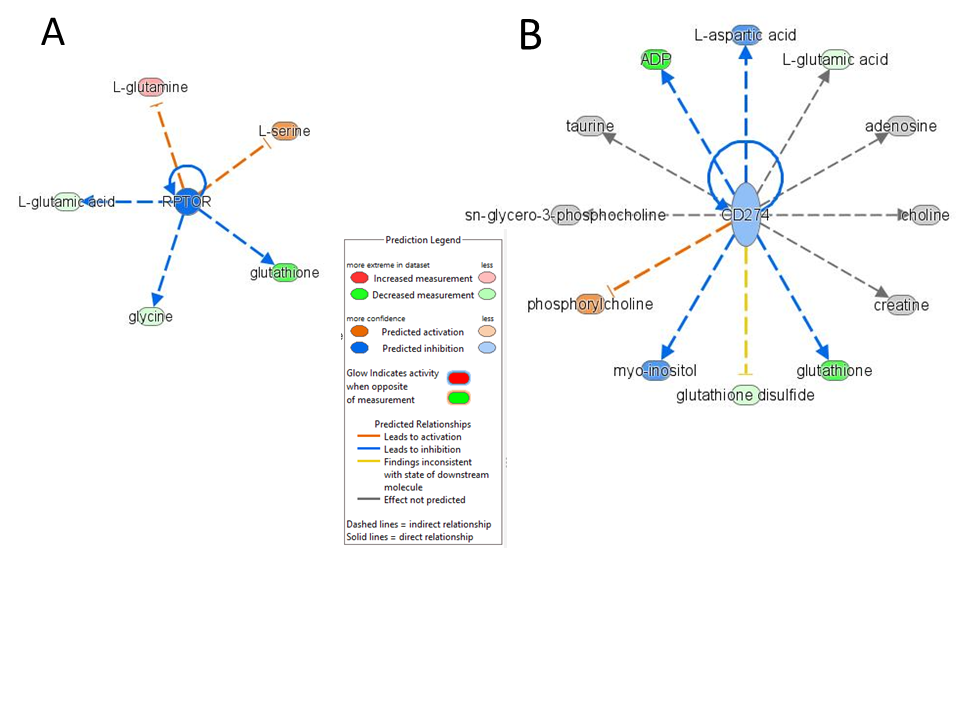


**Figure S5 – Networks of IPA's predicted significant upstream regulation potentially involved in the difference between breeds.**

These networks focus on key regulators like A- RPTOR and B- CD274.
The pointed arrowheads represent the trend of activating relationships while ~~and~~ the blunt arrowheads represent the trend of inhibitory relationships drawn from the available IPA literature. The blue, orange, and yellow colors indicate a predicted trend of inhibition, an activation, or an inconsistent finding of the relationship, respectively. The gray color indicates metabolites that were identified in the endometrium and did not vary between LW and MS.


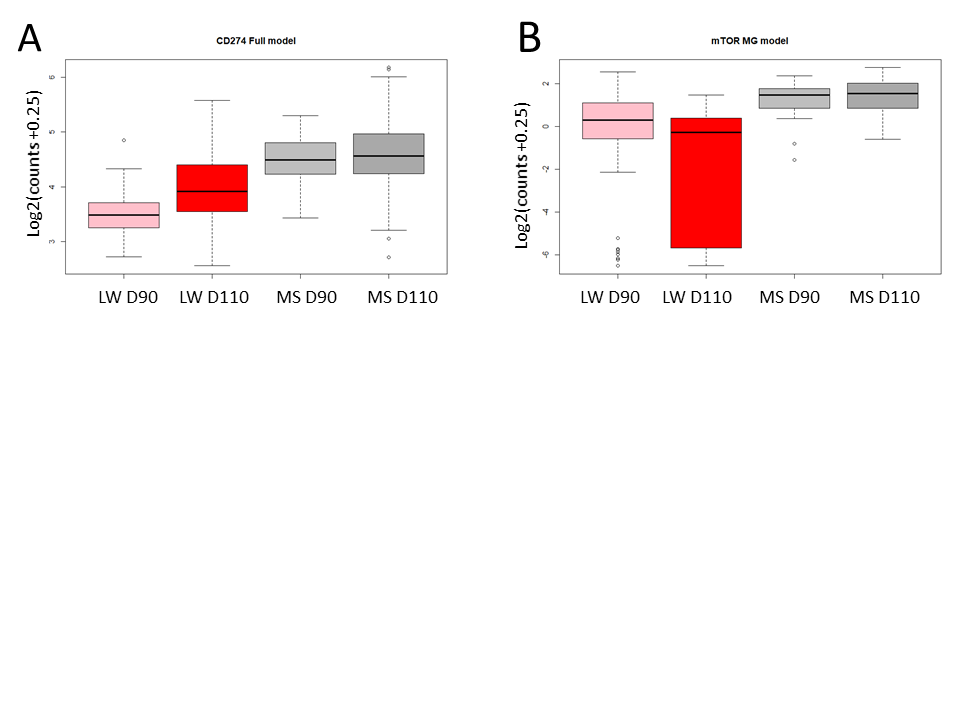


**Figure S6 - Gene expression of the top regulators**

Purple, red, light grey, and dark grey colors represent LW at D90, LW at D110, MS at D90, and MS at D110, respectively.


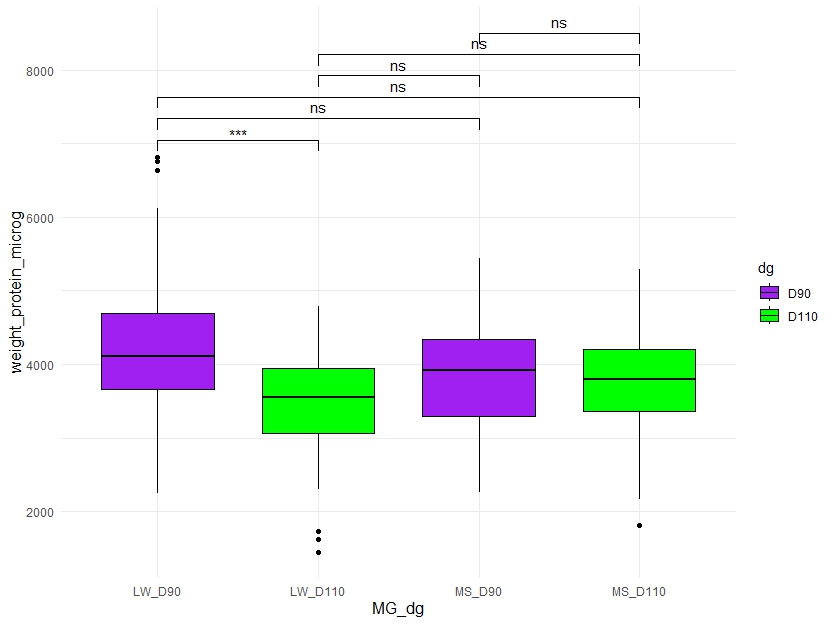


**Figure S7 – Protein content of endometrium tissue**

The protein content is expressed in µg of protein /mg of tissue. Significant change was performed using a Wilcoxon test and reported in the figure.


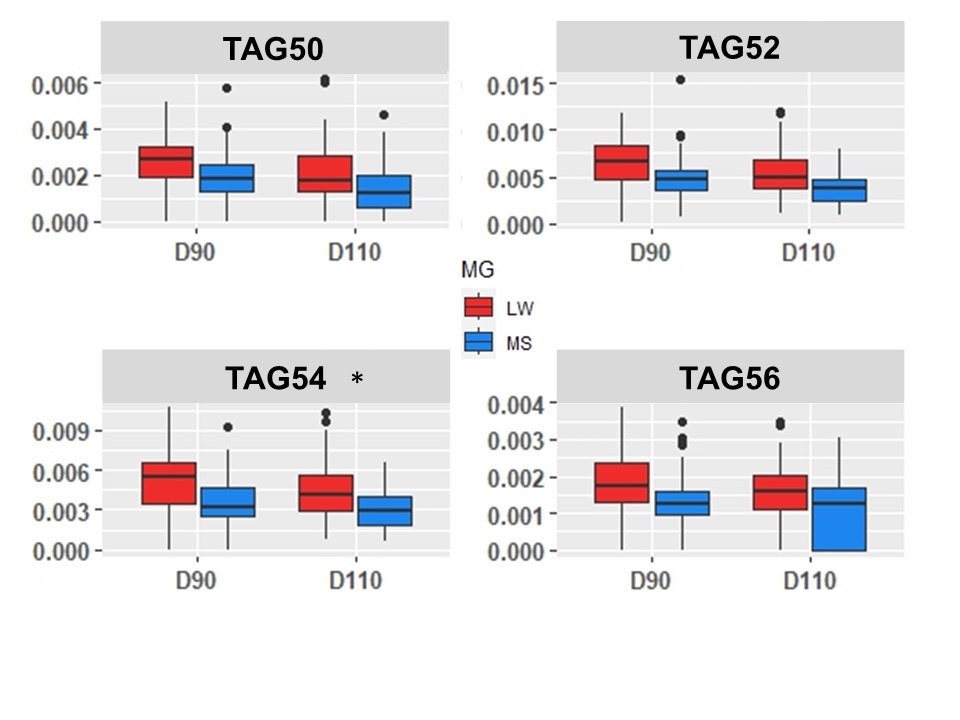


**Figure S8 – Triglycerides relative abundance**

Relative abundance for triglycerides that showed significant difference (TAG52 and TAG54; adjusted *p*-value<0.05) or an adjusted *p*-value<0.1 (TAG50 and TAG56) for DG + MG (additive effects).

**Table S1– Significant canonical pathways of endometrial metabolites affected by DG (adjusted *p*-value <0.05)**

Green: decreased abundance during the gestation, so abundancy at D90 > abundancy at D110

Red: increase d abundance during the gestation, so abundancy at D90 < abundancy at D110

Black: detected in the endometrium of sows

Only the pathways with an adjusted *p*-value < 0.05 (-log10(adjusted *p*-value) > 1.3) and with positive Z-score or negative Z-score were selected.

| Ingenuity Canonical Pathways | -log10(adjusted *p*-value) |  | Ratio | Z-score | Molecules |
| --- | --- | --- | --- | --- | --- |
| Transport of bile salts and organic acids, metal ions and amine compounds | 1.3E01 |  | 2.46E-01 | 1.134 | betaine, choline, citric acid, creatinine, glycine, L-alanine, L-glutamine, L-isoleucine, L-lactic acid, L-leucine, L-phenylalanine, L-proline, L-serine, L-tyrosine, L-valine, succinic acid, taurine |
| Tryptophan catabolism | 6.11E00 |  | 3.08E-01 | 1.000 | L-alanine,L-glutamic acid,L-isoleucine,L-leucine,L-phenylalanine,L-tyrosine,L-valine,NAD |
| Phenylalanine and tyrosine metabolism | 6.17E00 |  | 3.2E-01 | 0.447 | glutathione,L-alanine,L-aspartic acid,L-glutamic acid,L-phenylalanine,L-tyrosine,methanol,NAD |
| tRNA Charging | 9.39E00 |  | 2.79E-01 | 0.000 | glycine,L-alanine,L-aspartic acid,L-glutamic acid,L-glutamine,L-isoleucine,L-leucine,L-phenylalanine,L-proline,L-serine,L-tyrosine,L-valine |
| Transport of inorganic cations/anions and amino acids/oligopeptides | 1.25E01 |  | 2.24E-01 | -0.378 | acetic acid,betaine,glycine,L-alanine,L-aspartic acid,L-cystine,L-glutamic acid,L-glutamine,L-isoleucine,L-lactic acid,L-leucine,L-phenylalanine,L-proline,L-serine,L-tyrosine,L-valine,taurine |
| Sulfur amino acid metabolism | 4.11E00 |  | 1.67E-01 | -1.000 | adenosine,betaine,glutathione,glutathione disulfide,L-glutamic acid,L-serine,NAD ,taurine |
| G alpha (i) signalling events | 2.76E00 |  | 1.36E-01 | -1.000 | adenosine,ADP,L-glutamic acid,L-lactic acid,succinic acid,UDP-D-glucose |
| Nucleotide catabolism | 2.08E00 |  | 7.53E-02 | -1.000 | 2'-deoxyadenosine,adenosine,ADP,hypoxanthine,IMP,L-alanine,NAD |
| Nucleotide salvage | 1.92E00 |  | 9.62E-02 | -1.000 | 2'-deoxyadenosine,adenosine,ADP,hypoxanthine,IMP |
| Transport of vitamins, nucleosides, and related molecules | 1.67E00 |  | 8.2E-02 | -1.000 | 2'-deoxyadenosine,adenosine,ADP,hypoxanthine,UDP-D-glucose |
| Phase II - Conjugation of compounds | 3.26E00 |  | 1.21E-01 | -1.342 | adenosine,ADP,glutathione,glycine,L-glutamic acid,L-glutamine,NAD ,UDP-D-glucose |
| Aspartate and asparagine metabolism | 3.03E00 |  | 2.17E-01 | -2.000 | acetic acid,L-aspartic acid,L-glutamic acid,L-glutamine,taurine |
| Glutamate and glutamine metabolism | 4.88E00 |  | 2.8E-01 |  | ADP,L-alanine,L-aspartic acid,L-glutamic acid,L-glutamine,L-proline,NAD |
| Purine Nucleotides De Novo Biosynthesis II | 4.35E00 |  | 2.33E-01 |  | ADP,glycine,IMP,L-aspartic acid,L-glutamic acid,L-glutamine,NAD |

**Table S2 – IPA predicted significant upstream regulators** **in endometrial metabolites** **for DG**

Green: decreased abundance during the gestation, so abundancy at D90 > abundancy at D110

Red: increase d abundance during the gestation, so abundancy at D90 < abundancy at D110

Black: detected in the endometrium of sows

Only the regulators with an adjusted *p*-value < 0.05 and with positive Z-score or negative Z-score were selected.

| Upstream Regulator | Molecule Type | Activation Z-score | *p*-value of overlap | Adjusted *p*-value | Target Molecules in Dataset |
| --- | --- | --- | --- | --- | --- |
| IL37 | cytokine | -1.982 | 4.41E-09 | 2.55E-07 | citric acid, glutathione disulfide,glycine,hypoxanthine,IMP,L-phenylalanine,L-serine,succinic acid |
| HTT | transcription regulator | -1.408 | 2.00E-12 | 2.19E-10 | adenosine,choline,creatine,creatinine,glutathione,glycine,L-glutamic acid,myo-inositol,NAD+,sn-glycero-3-phosphocholine,succinic acid,taurine |
| HNF1B | transcription regulator | -1.342 | 2.62E-10 | 1.91E-08 | creatine,glutathione,glycine,L-aspartic acid,L-tyrosine,phosphorylcholine,succinic acid,taurine |
| D-glucose | chemical - endogenous mammalian | -1.231 | 3.53E-07 | 1.07E-05 | ADP,citric acid,creatine,glutathione,glutathione disulfide,L-aspartic acid,L-glutamic acid,pyrrolidonecarboxylic acid,sorbitol,succinic acid |
| 2-deoxyglucose | chemical drug | -1.036 | 5.04E-06 | 1.04E-04 | adenosine,glutathione,glutathione disulfide,hypoxanthine,IMP |
| HPD | enzyme | -1.000 | 4.60E-06 | 9.99E-05 | L-glutamic acid,L-phenylalanine,L-tyrosine,succinic acid |
| CHKA | kinase | -1.000 | 1.03E-15 | 3.76E-13 | adenosine,ADP,choline,creatine,glutathione,glutathione disulfide,L-aspartic acid,L-glutamic acid,myo-inositol,phosphorylcholine,sn-glycero-3-phosphocholine,taurine |
| PDPK1 | kinase | -0.555 | 2.81E-09 | 1.71E-07 | creatine,glutathione,L-aspartic acid,L-phenylalanine,L-tyrosine,taurine |
| FDX1 | transporter | -0.218 | 7.60E-05 | 1.13E-03 | hypoxanthine,IMP,L-glutamic acid,L-serine,taurine |
| MYC | transcription regulator | -0.218 | 6.10E-07 | 1.72E-05 | ADP,citric acid,creatinine,glutathione,glutathione disulfide,glycine,L-glutamic acid,succinic acid |
| ethanol | chemical - endogenous mammalian | -0.163 | 1.81E-02 | 4.53E-02 | adenosine,glutathione,L-glutamic acid,L-glutamine,taurine |
| lipopolysaccharide | chemical drug | -0.152 | 4.65E-06 | 9.99E-05 | ADP,citric acid,creatine,creatinine,glutathione,glutathione disulfide,L-aspartic acid,L-glutamic acid,L-glutamine,pyrrolidonecarboxylic acid,succinic acid,UDP-D-glucose |
| AQP7 | transporter | -0.124 | 8.31E-09 | 3.79E-07 | creatine,creatinine,glutathione,glutathione disulfide,L-aspartic acid,L-cystine,L-glutamic acid,L-proline,pyrrolidonecarboxylic acid |
| CD274 | transmembrane receptor | 0.000 | 3.69E-16 | 2.02E-13 | adenosine,ADP,choline,creatine,glutathione,glutathione disulfide,L-aspartic acid,L-glutamic acid,myo-inositol,phosphorylcholine,sn-glycero-3-phosphocholine,taurine |
| CPT1B | enzyme | 0.000 | 5.67E-12 | 5.18E-10 | adenosine,glycine,hypoxanthine,IMP,L-aspartic acid,L-glutamine,L-isoleucine,L-leucine,L-phenylalanine,L-tyrosine,L-valine |
| MMP11 | peptidase | 0.132 | 2.24E-15 | 6.15E-13 | choline,creatine,ethanolamine,glycine,L-glutamic acid,L-phenylalanine,L-tyrosine,phosphorylcholine,sn-glycero-3-phosphocholine,taurine |
| SIRT5 | enzyme | 0.152 | 7.05E-08 | 2.27E-06 | glutathione,glycine,L-glutamic acid,L-phenylalanine,L-proline,L-tyrosine |
| OGT | enzyme | 0.447 | 1.31E-08 | 5.33E-07 | glutathione,glycine,L-aspartic acid,L-glutamic acid,L-phenylalanine,L-proline,L-serine,L-tyrosine |
| CA9 | enzyme | 0.447 | 2.07E-16 | 2.02E-13 | glycine,L-alanine,L-aspartic acid,L-glutamine,L-isoleucine,L-leucine,L-phenylalanine,L-proline,L-serine,L-tyrosine,L-valine |
| CD40 | transmembrane receptor | 1.000 | 1.47E-06 | 3.94E-05 | ADP,creatine,hypoxanthine,IMP,L-phenylalanine,L-proline,L-tyrosine |
| IL10 | cytokine | 1.067 | 1.02E-04 | 1.45E-03 | creatinine,glutathione,L-glutamic acid,succinic acid |
| LDL | complex | 1.109 | 2.40E-06 | 6.27E-05 | citric acid,glutathione,L-aspartic acid,L-glutamic acid,L-lactic acid,phosphorylcholine,succinic acid |

**Table S3 – Canonical pathways for MG**

Green: decreased abundance between breeds (LW/MS), so abundancy in LW > abundancy in MS

Red: increased abundance between breeds (LW/MS), so abundancy in LW < abundancy in MS

Black: detected in the endometrium of sows

Only the pathways with an adjusted *p*-value < 0.05(-log10(adjusted *p*-value) > 1.3) and with positive Z-score or negative Z-score were selected.

| Ingenuity Canonical Pathways | -log(adjusted *p*-value) | Ratio | Z-score | Molecules |
| --- | --- | --- | --- | --- |
| Ferroptosis Signaling Pathway | 2.66E00 | 1.67E-01 | 1.000 | glutathione,glutathione disulfide,glycine,L-cystine,L-glutamic acid |
| Transport of bile salts and organic acids, metal ions and amine compounds | 1.3E01 | 2.46E-01 | 0.000 | betaine,choline,citric acid,creatinine,glycine,L-alanine,L-glutamine,L-isoleucine,L-lactic acid,L-leucine,L-phenylalanine,L-proline,L-serine,L-tyrosine,L-valine,succinic acid,taurine |
| Transport of inorganic cations/anions and amino acids/oligopeptides | 1.25E01 | 2.24E-01 | 0.000 | acetic acid,betaine,glycine,L-alanine,L-aspartic acid,L-cystine,L-glutamic acid,L-glutamine,L-isoleucine,L-lactic acid,L-leucine,L-phenylalanine,L-proline,L-serine,L-tyrosine,L-valine,taurine |
| tRNA Charging | 9.39E00 | 2.79E-01 | 0.000 | glycine,L-alanine,L-aspartic acid,L-glutamic acid,L-glutamine,L-isoleucine,L-leucine,L-phenylalanine,L-proline,L-serine,L-tyrosine,L-valine |
| Glutathione Biosynthesis | 3.57E00 | 0.5 | 0.000 | ADP,glutathione,glycine,L-glutamic acid |
| 5-aminoimidazole Ribonucleotide Biosynthesis I | 2.76E00 | 2.67E-01 | -1.000 | ADP,glycine,L-glutamic acid,L-glutamine |
| Phenylalanine Degradation IV (Mammalian, via Side Chain) | 3.26E00 | 2.5E-01 | -1.000 | glycine,L-glutamic acid,L-glutamine,L-phenylalanine,NAD |
| Purine Nucleotides De Novo Biosynthesis II | 4.35E00 | 2.33E-01 | -1.000 | ADP,glycine,IMP,L-aspartic acid,L-glutamic acid,L-glutamine,NAD |
| Phase II - Conjugation of compounds | 3.26E00 | 1.21E-01 | -1.342 | adenosine,ADP,glutathione,glycine,L-glutamic acid,L-glutamine,NAD ,UDP-D-glucose |
| Interconversion of nucleotide di- and triphosphates | 2.01E00 | 1.06E-01 | -1.342 | ADP,glutathione,glutathione disulfide,L-glutamic acid,L-glutamine |
| TP53 Regulates Metabolic Genes | 3.03E00 | 2.17E-01 | -1.342 | ADP,glutathione,glutathione disulfide,L-glutamic acid,L-glutamine |
| Selenoamino acid metabolism | 4.13E00 | 1.7E-01 | -2.000 | adenosine,ADP,glutathione,glutathione disulfide,glycine,L-alanine,L-serine,methanol |
| Arachidonic acid metabolism | 2.74E00 | 1.08E-01 | -2.236 | ADP,ethanolamine,glutathione,glutathione disulfide,glycine,L-glutamic acid,NAD |

**Table S4 – IPA predicted significant upstream regulators** **for maternal genotypes**

Green: decreased abundance between breeds (LW/MS), so abundancy in LW > abundancy in MS

Red: increased abundance between breeds (LW/MS), so abundancy in LW < abundancy in MS

Black: detected in the endometrium of sows

Only the regulators with an adjusted *p*-value < 0.05(-log10(adjusted *p*-value) > 1.3) and with positive Z-score or negative Z-score were selected.

| Upstream Regulator | Molecule Type | Predicted Activation State | Activation Z-score | P-value of overlap | Adjusted *p*-value | Target Molecules in Dataset |
| --- | --- | --- | --- | --- | --- | --- |
| RPTOR | other | Inhibited | -2.000 | 5.95E-07 | 1.72E-05 | glutathione,glycine,L-glutamic acid,L-glutamine,L-serine |
| liraglutide | biologic drug |  | -1.342 | 1.02E-11 | 8.63E-10 | adenosine,citric acid,creatinine,glycine,L-aspartic acid,L-glutamic acid,L-phenylalanine,L-proline,L-serine,L-tyrosine,myo-inositol,pyrrolidonecarboxylic acid,succinic acid,taurine |
| MYC | transcription regulator |  | -0.647 | 6.10E-07 | 1.72E-05 | ADP,citric acid,creatinine,glutathione,glutathione disulfide,glycine,L-glutamic acid,succinic acid |
| CD274 | transmembrane receptor |  | -0.577 | 3.69E-16 | 2.02E-13 | adenosine,ADP,choline,creatine,glutathione,glutathione disulfide,L-aspartic acid,L-glutamic acid,myo-inositol,phosphorylcholine,sn-glycero-3-phosphocholine,taurine |
| SIRT5 | enzyme |  | -0.152 | 7.05E-08 | 2.27E-06 | glutathione,glycine,L-glutamic acid,L-phenylalanine,L-proline,L-tyrosine |
| OGT | enzyme |  | 0.000 | 1.31E-08 | 5.33E-07 | glutathione,glycine,L-aspartic acid,L-glutamic acid,L-phenylalanine,L-proline,L-serine,L-tyrosine |
| D-glucose | chemical - endogenous mammalian |  | 0.554 | 3.53E-07 | 1.07E-05 | ADP,citric acid,creatine,glutathione,glutathione disulfide,L-aspartic acid,L-glutamic acid,pyrrolidonecarboxylic acid,sorbitol,succinic acid |
| IL37 | cytokine |  | 0.577 | 4.41E-09 | 2.55E-07 | citric acid,glutathione disulfide,glycine,hypoxanthine,IMP,L-phenylalanine,L-serine,succinic acid |
| CHKA | kinase |  | 1.414 | 1.03E-15 | 3.76E-13 | adenosine,ADP,choline,creatine,glutathione,glutathione disulfide,L-aspartic acid,L-glutamic acid,myo-inositol,phosphorylcholine,sn-glycero-3-phosphocholine,taurine |
| deoxycholate | chemical - endogenous mammalian |  | 1.981 | 2.79E-07 | 8.73E-06 | ADP,citric acid,glycine,L-aspartic acid,L-glutamic acid,L-proline,L-serine,UDP-D-glucose |
